# Fat and lean mass predict time to hospital readmission or mortality in children treated for complicated severe acute malnutrition in Zimbabwe and Zambia

**DOI:** 10.1017/S0007114522004056

**Published:** 2022-12-27

**Authors:** Mutsa Bwakura-Dangarembizi, Cherlynn Dumbura, Deophine Ngosa, Florence D. Majo, Joe D. Piper, Jonathan P. Sturgeon, Kusum J. Nathoo, Beatrice Amadi, Shane Norris, Bernard Chasekwa, Robert Ntozini, Jonathan C. Wells, Paul Kelly, Andrew J. Prendergast, for the HOPE-SAM Study Team

**Affiliations:** 1 University of Zimbabwe, Faculty of Medicine and Health Sciences, Harare, Zimbabwe; 2 Zvitambo Institute for Maternal and Child Health Research, Harare, Zimbabwe; 3 University of Witwatersrand, Johannesburg, South Africa; 4 Tropical Gastroenterology and Nutrition Group, University of Zambia, Lusaka, Zambia; 5 Blizard Institute, Queen Mary University of London, London, UK; 6 Population Policy and Practice Research and Teaching Department, UCL Great Ormond Street Institute of Child Health, London, UK

**Keywords:** Lean mass, Fat mass, Mortality, Hospitalisation, Severe acute malnutrition, HIV

## Abstract

HIV and severe wasting are associated with post-discharge mortality and hospital readmission among children with complicated severe acute malnutrition (SAM); however, the reasons remain unclear. We assessed body composition at hospital discharge, stratified by HIV and oedema status, in a cohort of children with complicated SAM in three hospitals in Zambia and Zimbabwe. We measured skinfold thicknesses and bio-electrical impedance analysis (BIA) to investigate whether fat and lean mass were independent predictors of time to death or readmission. Cox proportional hazards models were used to estimate the association between death/readmission and discharge body composition. Mixed effects models were fitted to compare longitudinal changes in body composition over 1 year. At discharge, 284 and 546 children had complete BIA and skinfold measurements, respectively. Low discharge lean and peripheral fat mass were independently associated with death/hospital readmission. Each unit *Z*-score increase in impedance index and triceps skinfolds was associated with 48 % (adjusted hazard ratio 0·52, 95 % CI (0·30, 0·90)) and 17 % (adjusted hazard ratio 0·83, 95 % CI (0·71, 0·96)) lower hazard of death/readmission, respectively. HIV-positive *v*. HIV-negative children had lower gains in sum of skinfolds (mean difference −1·49, 95 % CI (−2·01, −0·97)) and impedance index *Z*-scores (–0·13, 95 % CI (−0·24, −0·01)) over 52 weeks. Children with non-oedematous *v*. oedematous SAM had lower mean changes in the sum of skinfolds (–1·47, 95 % CI (−1·97, −0·97)) and impedance index *Z*-scores (–0·23, 95 % CI (−0·36, −0·09)). Risk stratification to identify children at risk for mortality or readmission, and interventions to increase lean and peripheral fat mass, should be considered in the post-discharge care of these children.

Malnutrition remains a global health challenge contributing to nearly 50 % of all deaths in children below the age of 5 years^(1)^. Severe acute malnutrition (SAM), defined as a weight-for-height *Z*-score (WHZ) <–3, mid-upper arm circumference (MUAC) <115 mm and/or bilateral nutritional oedema, has particularly high mortality when associated with clinical complications requiring hospitalisation. SAM is classified into oedematous and non-oedematous forms, and treatment success is defined by a target WHZ, MUAC or resolution of oedema, depending on the criteria initially used for classification^(2,3)^. Children hospitalised for complicated SAM remain at high risk for mortality in the year following hospital discharge. In a cohort of children in southern Africa, we recently showed that non-oedematous SAM, ongoing SAM at the time of discharge, cerebral palsy and HIV infection were independent predictors of mortality^(4)^.

None of the anthropometric measures used to define SAM estimates the restoration of associated physiological and functional deficits^(5,6)^. Body composition, measured as fat mass (FM) and lean mass (LM), has been shown to predict clinical outcomes in conditions such as cancer^(7,8)^, chronic kidney disease^(9)^ and sickle cell disease in children and adolescents^(10)^. LM comprises skeletal muscle, the skeleton, soft lean tissues and vital organs, while FM is distributed into central and peripheral fat stores^(11,12)^. Loss of LM has been associated with decreased survival, worse clinical outcomes, increased rate of infections, complications, duration of hospitalisation and prolonged recovery in adults with chronic diseases,^(13,14,15)^ and similar data are emerging for children^(16)^.

Interest in measuring body composition in undernourished children and exploring the long-term consequences for the development of non-communicable diseases has increased over the past few decades as mortality from SAM falls and children survive into adulthood^(17)^. Wasting is associated with deficits in both LM and FM^(18)^ and body composition can be used to evaluate physiological recovery following SAM^(3,19)^. However, there are few published data on the relationship between body composition and mortality, readmission and growth recovery in children managed for SAM. Assessing body composition may improve our understanding of underlying mechanistic pathways, recovery of physiological function and how to optimise clinical outcomes^(6)^.

We measured body composition in a longitudinal cohort of children followed for 52 weeks after discharge from hospitals in Zambia and Zimbabwe following management of complicated SAM. We hypothesised that low LM and FM would identify children most at risk of death or hospital readmission following hospital discharge for complicated SAM, and that body composition differences by HIV and oedema status may partly explain the differential long-term outcomes between children^(4,20)^.

## Methods

### Study design

This cohort was enrolled in the Health Outcomes, Pathogenesis and Epidemiology of Severe Acute Malnutrition (HOPE-SAM) study, which has been described in detail elsewhere^(21)^. In brief, HOPE-SAM was a longitudinal observational cohort of children under 5 years of age hospitalised for complicated SAM at three tertiary referral hospitals in Zambia and Zimbabwe between August 2016 and March 2018^(22)^. The unit at the University Teaching Hospital in Lusaka Zambia had a dedicated standalone facility that provided care for children hospitalised with complicated SAM and in Zimbabwe children received care in the general paediatric wards under different medical teams. Parents of children hospitalised for complicated SAM were sensitised about the study within 24 h of admission and enrolled upon consenting. Body composition and anthropometric measurements were done on admission during hospitalisation, upon discharge and during the post-discharge period. Follow-up care in both countries was provided by the HOPE SAM study teams. The protocol, standard operating procedures and pre-specified analysis plan for this manuscript are available at https://osf.io/29uaw/. This study was conducted according to the guidelines laid down in the Declaration of Helsinki and all procedures involving human subjects were approved by the University of Zambia Biomedical Research Ethics Committee (010-02-16), and the Medical Research Council of Zimbabwe (MRCZ/A/2044). The ethics committee of Queen Mary University of London provided an advisory review. Written informed consent was obtained from the participants’ parents/caregivers.

The main objectives of the current analysis were to: (1) determine the relationship between body composition and time to death or first hospital readmission over 52 weeks of follow-up; (2) compare body composition at discharge and over 52 weeks of follow-up between children with and without HIV infection and between children with non-oedematous *v*. oedematous SAM and (3) identify groups of children who share common body composition characteristics to determine whether the highest-risk children can be identified at the time of hospital discharge.

### Study population

The HOPE-SAM study enrolled children admitted below 60 months of age into medical wards with complicated SAM, defined as WHZ < –3 and/or MUAC < 115 mm and/or the presence of nutritional oedema (in children above 6 months of age), and WHZ < –3 or nutritional oedema in those below 6 months, based on WHO criteria^(2)^. Children who had malignancy, who died prior to study enrolment or whose caregiver was not willing to learn the child’s HIV status were not included in the study.

### Study procedures and follow-up

The study procedures have been described previously^(22)^. Baseline demographics, clinical, household and caregiver data were collected on paper case report forms and double data entered into a study database. Inpatient care was provided by ward staff separate from the research team, using WHO country-adapted guidelines for the management of complicated SAM^(2,23)^; however, progress during hospitalisation was documented daily by a study physician, with advice provided to the clinical teams on management. Children were seen in a dedicated study clinic at 2, 4, 12, 24 and 48 weeks after discharge. Details of hospital readmission and time to readmission were verified during study visits from children’s hand-held cards. Mortality was determined through telephone calls and home visits for those who defaulted follow-up. Each study visit had a window period around the target date. The 48-week visit window was deliberately wide (up to 72 weeks) to minimise loss to follow-up at the end of the study; since 86·4 % of data were collected by 52 weeks post-discharge, all analyses were censored at 52 weeks.

### Anthropometry measures

The following measurements were done on hospital admission, discharge and at 2, 4, 12, 24 and 48 weeks post-discharge: weight, height and MUAC using standardised methods^(4)^; body composition measured by bio-electrical impedance analysis (BIA) and triceps, subscapular and supra-iliac skinfold thickness.

### Bioelectrical impedance analysis measurements

Body composition was measured by whole body (wrist ankle) BIA using a BodyStat 1500 MD machine (BodyStat Ltd., Douglas, Isle of Man). BIA is a non-invasive method used to estimate body composition parameters. The technique involves placing two electrodes each on different parts of the body (e.g. the hand and feet) and passing a painless alternating electrical current through the body, which measures the opposition or impedance (Z) to current flow in Ohms through various tissues at 5 kHz (Z5) and 50 kHz (Z50)^(24)^. Impedance comprises resistance (R) which is the opposition of tissue to the flow of current and reactance (X_c_), which reflects the capacitative losses caused by cell membranes. Tissues containing large amounts of fluid and electrolytes have high conductivity and therefore low impedance, while fat and bone have low conductivity and high impedance^(24)^. Phase angle (PA) is a marker of the quality and quantity of soft tissue mass^(25)^; a smaller PA suggests cell death, reduced cell integrity and breakdown in cell membrane permeability^(26)^. BIA measurements were repeated until two impedance measurements taken at 50 kHz were within 5 Ohms. Additional readings were taken if the impedance measurements had a difference of more than 5 Ohms. If it was not possible to obtain two acceptable readings after five tests, the procedure was abandoned. Outlying values (defined as R > 1600 Ω, R < 300 Ω, X_c_ > 500 Ω, or PA > 8°) were discarded as implausible. If any one of the readings for resistance, reactance, PA or impedance was missing or excluded at a particular time point, all the other readings for that time point were excluded.

From the raw data of R, Z50 and Xc, we obtained proxies for LM and lean mass index (LMI) (lean mass/height^2^). The impedance index (Ht^2^/Z) is strongly associated with LM in any population and in epidemiological analyses behaves almost identically to LM when calibration equations are not available^(27)^, though oedema reduces the strength of this association^(28)^. For correct body size, LM is often adjusted for the square of height (Ht^2^) to give LMI. This means that the reciprocal of impedance expressed as 1/Z in units of 1/ohms again behaves almost identically to LMI in epidemiological analyses^(27)^. Both of these proxies were used in the current analyses. PA was also analysed as an independent composite marker of LM quality and quantity^(25)^.

### Skinfold thickness measurements

Skinfold thickness measurements are a simple and inexpensive method of estimating the size of the subcutaneous fat depot, which in turn correlates strongly with total body fat^(29,30)^. Subscapular, triceps and suprailiac skinfolds were taken on the left side of the body to the nearest 0·2 mm using calipers (Holtain Ltd.). Triceps skinfolds provided a measure of peripheral fat, while subscapular and suprailiac skinfolds provided a measure of central fat. Total skinfold thickness was taken as the sum of subscapular, triceps and suprailiac skinfolds. Skinfold readings were measured in triplicate, and medians, means and standard deviations of the values generated. Skinfold measurements were converted to *Z*-scores using 2006 WHO standards; we created our own internal *Z*-scores for suprailiac skinfolds for which there are no WHO standards. Longitudinal values of skinfolds were assessed for plausibility over the follow-up period; measures that were out of range based on WHO standards were verified on source documents and excluded as implausible.

### Statistical methods

#### Survival analysis

The primary outcome for this analysis was a composite of mortality or time to first hospital readmission. Children were censored at the last known alive date or at week 52 after discharge. Cox proportional hazards models were used to estimate hazard ratios of death or hospital readmission with body composition variables as exposures, after adjusting for the following minimum set of covariates obtained from a Directed Acyclic Graph: age at discharge, sex, height-for-age *Z*-score (HAZ) and WHZ at discharge, HIV status, oedema at hospitalisation and maternal employment (online Supplementary Methods).

#### Longitudinal changes in body composition

Data were first checked for consistency and completeness. All available data from participant discharge to study discontinuation were summarised. Non-overlapping visit windows were used and the measurement closest to the study visit week was used for analyses. Mixed effects models were used to compare longitudinal skinfolds *Z*-scores and BIA data converted to internal *Z*-scores between children with and without HIV infection and between children with and without oedema at initial hospitalisation, using HIV or oedema as fixed effects and participant identifier as the random effect. Directed Acyclic Graph constructed using DAGitty version 3.0 were used to select the minimum set of covariates for adjustment (online Supplementary Methods). The minimum covariate set for HIV status as an exposure were birthweight, sex, age at discharge, oedema at initial hospitalisation, HAZ and WHZ at discharge, hospital readmission, toilet type and maternal employment. The minimum covariate set for oedema as an exposure were birthweight, HIV status, age at discharge, hospital readmission, maternal employment and toilet type. Coefficients and confidence intervals obtained from the univariable and multivariable mixed effects models were interpreted. Margins plots were used to show the change in body composition by HIV status and baseline oedema status over 52 weeks of follow-up.

#### Clustering analysis

We conducted hierarchical clustering to identify groups of children sharing common characteristics based on discharge body composition measurements and anthropometric factors previously associated with mortality in the same cohort (oedema status at initial hospitalisation, HAZ and WHZ at discharge)^(4)^. The number of clusters was identified using the Calinski-Harabasz stopping rule. Comparison of characteristics and outcomes of children across groups was made using logistic regression for categorical variables and linear regression for continuous variables. All analyses were conducted using STATA version 14.0 (StataCorp.).

## Results

### Study enrolment

Of 750 children enrolled from July 2016 to March 2018, 5 were excluded and 745 were followed in hospital; 649 were discharged to continue nutritional rehabilitation in the community. Forty-five children left the hospital before any measurements were done, leaving 604 children with discharge measurements (online Supplementary Fig. 1). Complete BIA and skinfolds data were collected in 334 and 563 children, respectively. Following data cleaning and removal of implausible values, 284 (47 %) children contributed complete BIA data and 546 (90·4 %) skinfolds measurements at hospital discharge. Baseline characteristics of the children with and without discharge BIA measurements were broadly similar, except for small differences in country, caregiver employment and anthropometry. Children with missing data had a marginally lower WHZ, but higher HAZ than those with available BIA data and no differences in weight-for-age *Z*-score or MUAC (online Supplementary Table 1).

### Discharge characteristics

We have previously shown that post-discharge mortality is higher in children living with HIV compared with those without HIV and in children with non-oedematous compared with oedematous SAM^(4)^. We therefore stratified discharge characteristics by HIV and oedema status. At discharge, HIV-positive compared with HIV-negative children were significantly older and were more likely to have non-oedematous SAM at admission and ongoing SAM at discharge. Anthropometry (weight-for-age *Z*-score, HAZ and MUAC), sum of skinfolds, impedance and LMI were significantly lower in HIV-positive compared with HIV-negative children at the time of hospital discharge. Children with oedematous SAM were significantly older, less likely to be HIV-positive and less likely to have ongoing SAM at the time of hospital discharge compared with those with non-oedematous SAM. Children with non-oedematous SAM were more stunted and had significantly less fat and LM than children with oedematous SAM (Table 1).

### Associations between discharge body composition and time to death or hospital readmission

In the adjusted Cox regression model (Table 2), both LM and peripheral FM were independently associated with mortality or readmission into hospital. Each *Z*-score increase in LMI and impedance index was associated with a 33 % (adjusted hazard ratio 0·67, 95 % CI (0·47, 0·98)) and 48 % (adjusted hazard ratio 0·52, 95 % CI (0·30, 0·90)) reduction in hazard of death or hospital readmission. Each *Z*-score increase in triceps skinfold was associated with a 17 % (adjusted hazard ratio 0·83, 95 % CI (0·71, 0·96)) reduction in death or readmission. By contrast, subscapular and suprailiac skinfolds and PA were not associated with death or time to readmission. We tested for the interaction of each body composition variable by HIV and oedema status and found evidence of interaction between LMI and oedema status only. Stratified analysis showed that among children with oedema, each unit increase in LMI was associated with a 61 % (adjusted hazard ratio 0·39, 95 % CI (0·20, 0·75)) reduction in the hazards of death or readmission; by contrast, there was no association between LMI and death/readmission in children without oedema. In summary, LM and peripheral FM were independently associated with hospital readmission.

We further analysed the components of the composite variable separately and found that readmission and not death were significantly associated with low LM and peripheral FM (online Supplementary Tables 2a and b).

### Body composition over 52 weeks of follow-up

**Fig. 1:**
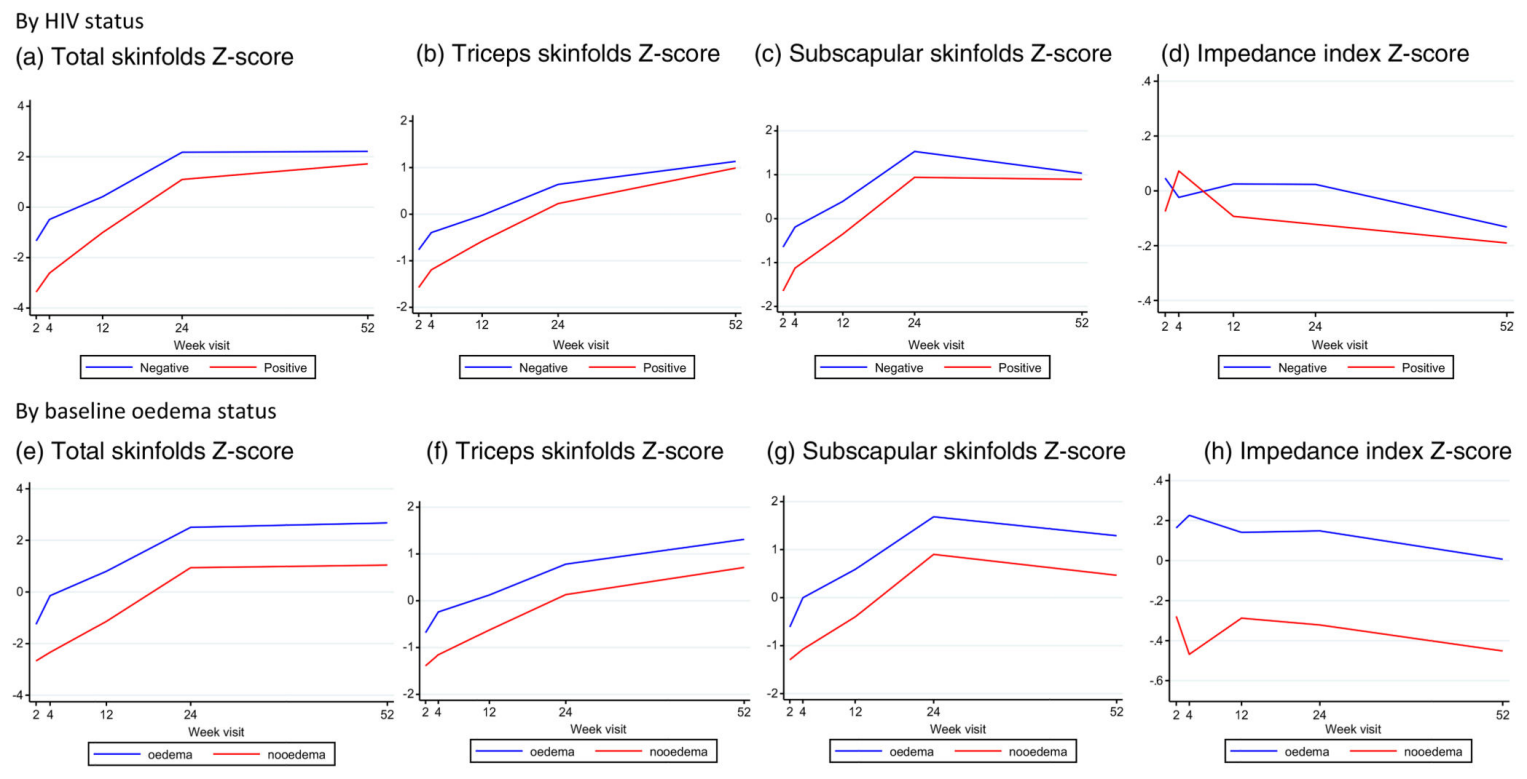
Margins plots of change in body composition over 52 weeks of follow-up stratified by HIV status and oedema status at initial hospitalisation.

Fig. 1 shows the change in skinfolds (representing FM) and impedance index (representing LM) over time stratified by HIV status and oedema status. Individual trajectories are shown as spaghetti plots in Supplementary Fig. 2. Over the 52 weeks of follow-up, all children showed a general increase in fat and LM. Triceps skinfolds (a measure of peripheral fat) increased throughout the period of follow-up; subscapular skinfolds (a measure of central fat) increased up to 24 weeks followed by a decline and sum of skinfolds (a marker of total fat) increased up to 24 weeks, then plateaued.

### Association between anthropometry and body composition with baseline oedema and HIV status

Table 3 shows the univariable and multivariable mixed effects analysis of the association between HIV and baseline oedema status, with anthropometry and body composition during follow-up. HIV-positive compared with HIV-negative children had lower gains in anthropometry, FM and LM over 52 weeks. Mean changes in MUAC (–0·44 cm, (95 % CI (−0·62, −0·25)) and HAZ (–0·32 *Z*-score (95 % CI (−0·60, –0·04)) were lower in HIV-positive compared with HIV-negative children. FM gains were lower in HIV-positive compared with HIV-negative children: −1·49 *Z*-scores (95 % CI (2·01, −0·97)) for total FM, −0·66 *Z*-scores (95 % CI (−0·91, −0·41)) for central FM, and −0·60 *Z*-scores (95 % CI (−083, −0·37)) for peripheral FM. Change in LM measured by impedance index was also lower in HIV-positive compared with HIV-negative children (–0·13 (95 % CI (−0·24, −0·01)) over 52 weeks.

Compared with children with oedema at the time of initial hospitalisation, mean changes in MUAC, WHZ and weight-for-age *Z*-score were significantly lower in children with non-oedematous SAM, while HAZ was not different between groups. Both FM and LM were significantly lower in children without oedema: −1·47 *Z*-score (95 % CI (−1·97, −0·97)) for total FM and −0·23 *Z*-scores (95 % CI (−0·36, −0·09)) for impedance index *Z*-score.

Taken together, whilst children showed overall gains in LM and FM and anthropometry during 52 weeks of follow-up, changes were significantly lower for HIV-positive children and for those with non-oedematous malnutrition.

### Clustering analysis

**Fig. 2:**
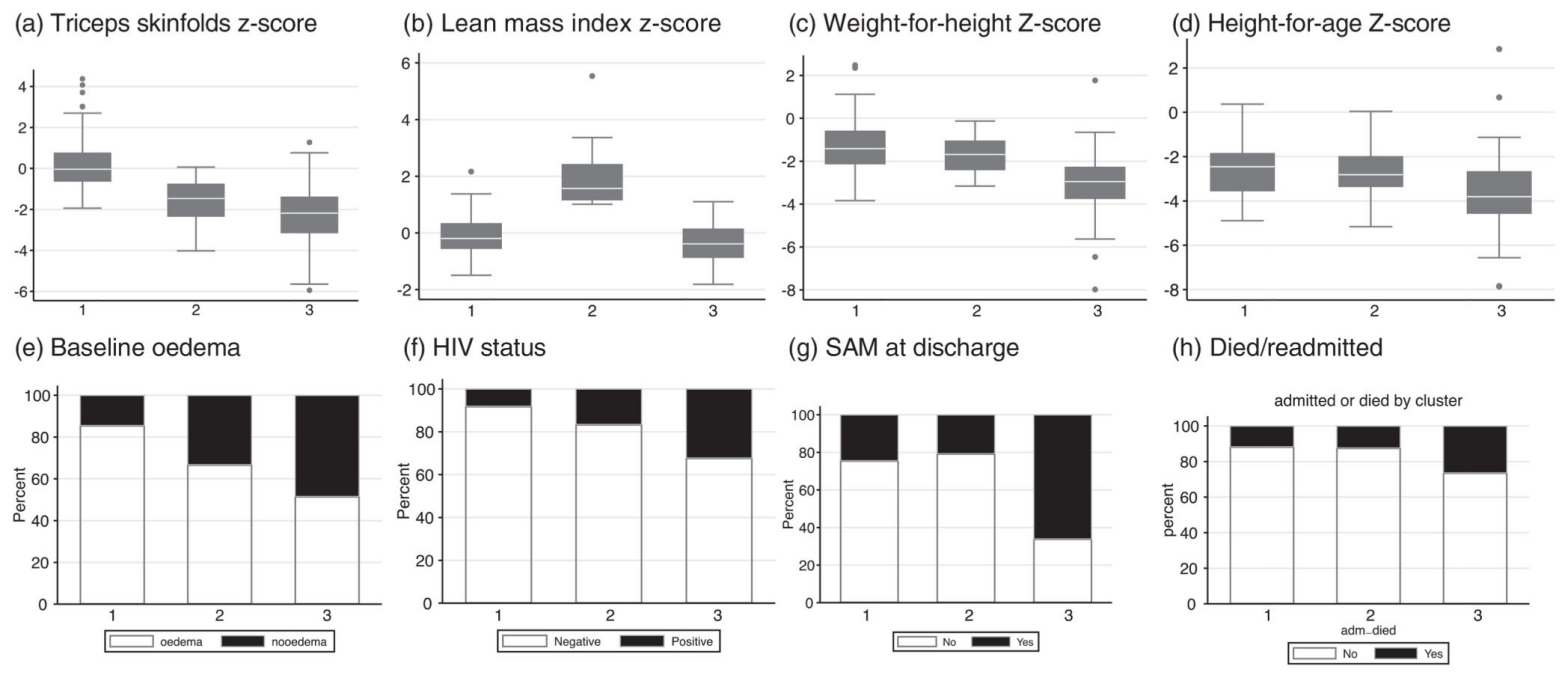
Characteristics of subgroups of children at discharge. Three groups were identified by hierarchical clustering; (Group 1 *n* 110, Group 2 *n* 24 and Group 3 *n* 136). The group characteristics are shown as proportions for each variable associated with poor outcomes (a. Triceps skinfolds *Z*-score, b. Lean mass index *Z*-score, c. WHZ, d. HAZ, e. baseline oedema, f. HIV status, g. SAM at discharge and H. composite outcome of died/readmitted). WHZ, weight-for-height *Z*-score; HAZ, height-for-age *Z*-score; SAM, severe acute malnutrition.

Hierarchical clustering based on body composition and anthropometry identified three groups of children at the time of hospital discharge, group 1 (*n* 110, 40·7 %), group 2 (*n* 24, 8·9 %) and group 3 (*n* 136, 50·4 %), whose characteristics were strongly associated with time to readmission or death (Fig. 2; online Supplementary Table 3, online Supplementary Fig. 3). Group 1 children had the highest proportion of oedema at hospitalisation (85·5 %), the highest FM and the lowest HIV infection rates. Group 2 had the highest LM compared with the other groups. Group 3 had the lowest FM and LM, WHZ and HAZ, the highest proportion of HIV-positive children (32·4 %) and ongoing SAM at the time of hospital discharge (66·2 %) compared with groups 2 and 3.

While group 1 had the highest FM and group 2 had the highest LM, the two groups had similar WHZ, HAZ, rates of HIV positivity and SAM at discharge. Interestingly, the odds of death or death or readmission were similar between groups 1 and 2, suggesting that there may be different pathways to survival involving the preservation of FM and LM, respectively. Overall, compared with group 1, children in group 3 had a 2·7-fold increased odds of death or readmission to hospital (adjusted OR 2·7 (95 % CI (1·3, 5·4)) after adjusting for age (online Supplementary Table 3).

## Discussion

In this cohort of children treated for complicated SAM and discharged from three hospitals in southern Africa, we show that body composition, in addition to anthropometry, is an important determinant of mortality or hospital readmission in the year following hospital discharge. Specifically, low LM and peripheral FM (using BIA and skinfold measurements) were independently associated with the composite outcome of death or readmission. Furthermore, a distinct phenotypic group of children at the highest risk for mortality and hospital readmission were characterised by particularly low WHZ, HAZ, LM and FM, high prevalence of HIV infection and ongoing SAM at the time of discharge. Collectively, these findings highlight the importance of considering body composition in the assessment of nutritional recovery and risk stratification of children with SAM in order to optimise post-discharge outcomes.

The most important clinical outcomes in the year post-discharge from the hospital are readmission or death. In this study, we showed that greater peripheral FM and LM were associated with reduced risk of readmission or death. Clinical studies in adults have shown that higher muscle mass is a major determinant of survival independent of body mass index^(31)^. Children have a lower muscle mass in relation to body weight compared with adults, and those with complicated SAM stand to suffer more from acute muscle wasting resulting from food insufficiency, infection and inflammation. Early studies on nutritional status and muscle mass showed that muscle mass was an important determinant of survival^(32)^, but few studies have evaluated LM in children with SAM. Here, we confirm that greater LM at discharge from the hospital is associated with reduced mortality or hospital readmission, regardless of HIV status, although the relationship between LM and clinical outcomes appeared to be confined to children with oedema. Only higher triceps skin-fold was associated with mortality or readmission, suggesting that the pattern of fat loss or preservation is important^(18)^. Fat is used as a metabolic fuel during periods of food deprivation, and this is reflected in peripheral fat loss. During complicated SAM, which is characterised by infection and inflammation, leptin from central fat stores plays a role in stimulating the immune system through lymphopoiesis^(33)^ and upregulation of the innate and adaptive immune response^(34)^. Advances in the measurement of FM and LM in malnourished children are set to provide more insight into monitoring growth and development, disease progression and response to treatment^(10)^.

We have previously shown that oedematous SAM accounted for nearly two-thirds of children hospitalised for complicated SAM in this cohort and that those with no oedema had twice the mortality risk 1 year post-discharge^(4)^. Children with non-oedematous SAM had slower anthropometric recovery and gained less FM and LM over the year following hospital discharge. Children hospitalised with oedema tend to recover anthropometric parameters faster than wasted children whose condition is more likely to have been longstanding. Non-oedematous SAM is characterised by loss of subcutaneous fat and muscle wasting, which takes a longer time to recover, in contrast to oedematous SAM in which subcutaneous fat and muscle mass are better preserved. Change in LM was significantly lower in children with non-oedematous SAM, suggesting that they take longer to recover from the insult of infection, inflammation and food deprivation. Results for PA were null, indicating that the gross amount of lean tissue, rather than more subtle markers of membrane quality, is important for survival.

There are sparse data^(18,35)^ on body composition in children recovering from complicated SAM. In this longitudinal study of under-five children in Zambia and Zimbabwe, we showed that both FM and LM were significantly lower in HIV-positive compared with HIV-negative children at the time of hospital discharge. Weight loss in HIV-infected persons affects both lean and fat tissue and remains a significant problem even in the era of combination antiretroviral therapy^(36)^. While there was a general increase in fat over 1 year of follow-up, the mean changes in regional and total fat were significantly lower in HIV-positive compared with HIV-negative children. These findings differ from a study on nutritional recovery among HIV-positive and HIV-negative children with SAM from Malawi, which showed similar improvements in triceps (peripheral fat) and subscapular (central fat) skinfolds over 4 months of follow-up^(37)^. The difference between studies may be because the Malawi study enrolled children with uncomplicated SAM, whereas we enrolled children discharged from the hospital after the management of complicated SAM. The additional burden of infection, inflammation and metabolic derangements in children with complicated SAM is likely to exert additional nutrient demands during the recovery period and may fundamentally alter body composition parameters. Studies have shown that the additional metabolic demands for amino acids to support the generation of acute phase proteins and an adaptive immune response exerts a catabolic effect, particularly on skeletal muscle^(31,38)^, resulting in loss of LM.

We were able to define three phenotypic groups based on anthropometry and body composition, with differences in mortality or readmission 1 year after discharge from the hospital. The group with the worst anthropometry, body composition markers, ongoing SAM and highest HIV infection rates had the highest odds of death or readmission compared with the other two groups. The similar odds of death or readmission among groups 1 and 2 whose main difference was FM and LM suggest that for a similar weight-for-height and height-for-age, the amount of LM or FM is associated with survival. The current classification of malnutrition that is based on body size may not have any link with the physiological changes that contribute to poor outcomes in these children. Children with complicated SAM have disrupted metabolic, organ and immunological function, and consideration should be given to using more physiological measures for risk stratification and development of interventions that restore healthy function.

The main strength of this study is that we had a large cohort that was followed longitudinally from hospital discharge to 52 weeks, in a setting of high HIV prevalence, and used rarely deployed techniques to measure body composition at the time of discharge. Our high outcome ascertainment for death and readmission due to close follow-up of this cohort strengthens our inferences regarding body composition and clinical outcomes. Limitations of this study include the inability to obtain bioelectrical impedance data on more than half of the participants. While BIA is a non-invasive and technically straightforward assessment of body composition, it requires cooperation from the child, who must be relaxed and still; this was not always easy, particularly at the time of discharge from the hospital. Nevertheless, the sample of children with BIA measurements had similar discharge characteristics to those without measurements, and their rates of mortality and readmission were similar. We were not able to determine the exact FM and fat-free mass, which requires isotope calibration against a reference standard; however, our proxy markers of impedance index and LMI provided robust relative measures of LM that behave similarly to calibrated values in epidemiological analyses. Another potential limitation is survivor bias, where the sickest children (including those who were living with HIV) died early and had less opportunity to be readmitted to the hospital.

### Conclusion

In summary, we show that body composition (in particular LM and peripheral fat) at the time of hospital discharge is important determinant of mortality and hospital readmission in children managed for complicated SAM. Complicated SAM is characterised by multiple deranged metabolic, immune and hormonal pathways. There is therefore need for interventions that address the inflammatory state that characterises complicated SAM during the convalescent period using therapeutic foods that reduce metabolic stress, modulate inflammation and increase nutrient bioavailability, to improve FM and LM recovery, particularly in the highest-risk children with non-oedematous SAM and HIV infection. In this cohort, anthropometry and body composition identified specific groups of children with poor outcomes over the year following discharge from the hospital. Body composition measurements reflect the physiological changes accompanying the response to treatment and recovery. Screening children prior to discharge using BIA and triceps skinfold measurement may be additional tools to identify those at risk for poor outcomes and would need more targeted interventions to improve their outcomes.

## Abbreviations

BIA: bioelectrical impedance analysis
FM: fat mass
HAZ: height-for-age *Z*-score
LM: lean mass
LMI: lean mass index
MUAC: mid-upper arm circumference
PA: phase angle
SAM: severe acute malnutrition
WHZ: weight-for-height *Z*-score.

## Supplementary Material

Supplementary Materials

## Figures and Tables

**Table 1:** Discharge anthropometry and body composition

	All *n* 604		HIV-positive *n* 121		HIV-negative *n* 483		P-value*	Oedema at Baseline *n* 391		No oedema at baseline *n* 213		P value†
	*n*	%	*n*	%	*n*	%		*n*	%	*n*	%	
Male	322/604	53·3	60/121	49·6	262/483	54·2	0·36	208/391	53·2	114/213	53·5	0·94
	Median	IQR	Median	IQR	Median	IQR		Median	IQR	Median	IQR	
Age, months	18·0	13·6, 22·8	20·2	15·4, 26·0	17·5	13·4, 22·1	<0.001	19·3	14·8, 23·3	15·7	11·2, 21·2	<0·001
	*n*	%	*n*	%	*n*	%		*n*	%	*n*	%	
Oedema at hospitalisation	391/604	64·7	63/121	52·1	328/483	67·9	0·001	n/a		n/a		n/a
HIV-positive	n/a		n/a		n/a			63/391	16·1	58/213	27·2	0·001
SAM at discharge	264/604	43·7	66/121	54·6	198/483	41·0	0·007	146/391	37·3	118/213	55·4	<0·001
	Mean	SD	Mean	SD	Mean	SD		Mean	SD	Mean	SD	
Anthropometry at discharge												
WHZ	−2·2	1·5	−2·4	1·4	−2·1	1·5	0·09	−1·9	1·4	−2·7	1·4	<0·001
WAZ	−3·3	1·6	−3·7	1·2	−3·2	1·7	0·006	−3·0	1·5	−4·0	1·5	<0·001
HAZ	−3·1	1·5	−3·4	1·2	−3·0	1·6	0·007	−2·9	1·5	−3·4	1·6	<0·001
MUAC, mm	123·0	15·7	116·9	13·5	124·6	15·9	<0·001	126·5	15·6	116·5	13·8	<0·001
MUAC *Z*-score	−2·4	1·61	−3·16	1·40	−2·23	1·61	<0·001	−2·10	1·61	−3·00	1·45	<0·001
Body composition at discharge												
Triceps skinfolds, mm	6·65	2·35	5·54	1·59	6·94	2·43	<0·001	6·98	2·54	6·01	1·78	<0·001
Triceps skinfolds *Z*-score	−1·19	1·69	−2·02	1·45	−0·97	1·68	<0·001	−0·93	1·71	−1·69	1·52	<0·001
Subscapular skinfolds, mm	5·21	1·97	4·46	1·70	5·35	2·00	<0·001	5·36	2·18	4·92	1·43	0·01
Subscapular skinfolds *Z*-score	−1·49	2·02	−2·10	2·02	−1·34	1·99	<0·001	−1·34	2·13	−1·79	1·76	0·01
Suprailiac skinfolds, mm	4·48	2·00	3·63	1·70	4·70	2·02	<0·001	4·65	2·07	4·16	1·82	0·006
Suprailiac skinfolds *Z*-score	0·002	1·00	0·11	1·01	−0·42	0·85	<0·001	0·08	1·03	−0·16	0·92	0·007
Sum of skinfolds, mm	16·39	5·07	14·10	4·29	17·02	5·07	<0·001	17·04	5·35	15·10	4·18	<0·001
Sum of skinfolds *Z*-score	0·008	1·00	−0·47	0·84	0·13	1·00	<0·001	0·14	1·05	−0·24	0·82	<0·001
Impedance index, cm^2^/Ω	0·63	0·19	0·59	0·17	0·64	0·20	0·07	0·67	0·18	0·56	0·19	<0·001
Impedance index *Z*-score	0·01	1·00	−0·20	0·90	0·06	1·02	0·07	0·22	0·95	−0·37	0·98	<0·001
Lean mass index, 1/Ω	11·73	2·43	10·97	2·12	11·92	2·47	0·008	11·96	2·32	11·30	2·56	0·03
Lean mass index *Z*-score	0·009	−0·11	−0·30	0·88	0·09	1·02	0·008	0·10	0·96	−0·17	1·06	0·03
Phase angle	3·26	1·27	3·31	1·34	3·25	1·26	0·77	3·23	1·26	3·33	1·30	0·54
Phase angle *Z*-score	0·001	−0·11	0·04	1·05	−0·01	1·00	0·77	−0·03	1·00	0·05	1·02	0·54

IQR, interquartile range; SAM, severe acute malnutrition; sd, standard deviation; WHZ, weight-for-height *Z*-score; WAZ, weight-for-age *Z*-score; HAZ, height-for-age *Z*-score; MUAC, mid-upper-arm circumference.

* *P* value comparing HIV-positive and HIV-negative groups.

† *P* value comparing children with and without oedema at initial hospitalisation.

Data are n/total (column %) unless otherwise stated. 1/Z (Ω) was used as a proxy for lean mass index.

**Table 2:** Cox regression model of discharge body composition factors associated with time to death or readmission

	*n*	Unadjusted HR	95% CI	P-value	Adjusted HR*	95 % CI	P-value
Bioimpedance analysis							
Lean mass index *Z*-score,	284	0·53	0·37, 0·75	0·002	0·62	0·41, 0·94	0·02
Impedance index *Z*-score	286	0·48	0·33, 0·71	<0·001	0·48	0·24, 0·83	0·01
Phase angle *Z*-score	296	1·03	0·79, 1·36	0·84	1·07	0·81, 1·43	0·63
Skin folds							
Triceps skinfold *Z*-score	557	0·72	0·63, 0·83	<0·001	0·77	0·64, 0·91	0·002
Subscapular skinfold *Z*-score	550	0·86	0·76, 0·97	0·01	0·89	0·79, 1·02	0·09
Suprailiac skinfold *Z*-score	563	0·75	0·58, 0·97	0·03	0·90	0·68, 1·19	0·46
Sum of skinfolds *Z*-score	551	0·63	0·47, 0·83	0·001	0·69	0·49, 0·98	0·04

HR, hazard ratio; WHZ, weight-for-height *Z*-score; HAZ, height-for-age *Z*-score.

* Multivariable models included a minimum adjustment set identified from a directed acyclic graph: age at discharge, sex, baseline oedema, WHZ, HAZ, HIV status and maternal employment.

**Table 3:** Univariable and multivariable mixed effects analysis of body composition and anthropometry over 52 weeks of follow-up

Variable	HIV-positive v HIV-negative				Non-oedematous v. oedematous			
	Unadjusted coefficient P value	95 % CI	Adjusted coefficient P value	95 % CI	Unadjusted coefficient P value	95 % CI	Adjusted coefficient P value	95 % CI
MUAC, cm	−0·81	−1·07, −0·54	−0·44	−0·62, −0·25	−1·09	−1·30, −0·88	−0·76	−0·98, −0 54
	<0·001		<0·001		<0·001		<0·001	
MUAC *Z*-score	−0·79	−1·05, −0·53	−0·44	−0·62, −0·25	−1·02	−1·23, −0·81	−0·70	−0·92, −0·48
	<0·001		<0·001		<0·001		<0·001	
Weight-for-height	−0·25	−0·51, 0·01	−0·11	−0·05, 0·27	−1·07	−1·27, −0·87	−0·89	−1·10, −0·67
*Z*-score	0·06		0·18		<0·001		<0·001	
Height-for-age	−0·39	−0·67, −0·12	−0·32	−0·60, −0·04	−0·51	−0·74, −0·28	−0·19	−0·44, 0·05
*Z*-score	0·005		0·03		<0·001		0·12	
Weight-for-age	−0·45	−0·78, −0·15	−0·04	−0·11, −0·18	−1·14	−1·38, −0·90	−0·77	−1·01, −0·52
*Z*-score	0·004		0·63		<0·001		<0·001	
Triceps skinfold	−0·86	−1·11, −0·62	−0·60	−0·83, −0·37	−0·85	−1·07, −0·64	−0·63	−0·85, −0·41
*Z*-score	<0·001		<0·001		<0·001		<0·001	
Subscapular skinfold *Z*-score	−0·93	−1·19, −0·67	−0·66	−0·91, −0·41	−0·91	−1·13, −0·69	−0·73	−1·00, −0·45
	<0·001		<0·001		<0·001		<0·001	
Suprailiac skinfold	−0·34	−0·48, −0·20	−0·30	−0·45, −0·15	−0·22	−0·34, −0·11	−0·11	−0·24, 0·02
*Z*-score	<0·001		<0·001		<0·001		0·09	
Total skinfolds	−2·08	−2·64, −1·52	−1·49	−2·01, −0·97	−1·95	−2·42, −1·48	−1·47	−1·97, −0·97
*Z*-score	<0·001		<0·001		<0·001		<0·001	
Lean mass index	−0·21	−0·37, −0·06	−0·11	−0·27, 0·04	−0·27	−0·40, −0·14	−0·23	−0·37, −0·08
*Z*-score	0·007		0·16		<0·001		0·002	
Impedance index	−0·31	−0·46, −0·16	−0·13	−0·24, −0·01	−0·37	−0·50, −0·24	−0·23	−0·36, −0·09
*Z*-score	<0·001		0·04		<0·001		0·002	
Phase angle	0·01	−0·15, 0·13	−0·001	−0·15, 0·15	−0·08	−0·04, 0·20	0·08	−0·06, 0·21
*Z*-score	0·90		1·0		0·21		0·25	

All values are mean differences (95% CI) from a multivariable mixed effects model, with HIV or oedema as fixed effects and participant identifier as the random effect.

Adjustment set for HIV status: sex, birthweight, age at discharge, oedema at hospitalisation, HAZ, WHZ, maternal employment, toilet type and hospital readmission.

Adjustment set for oedema status: birthweight, age at discharge, HIV status, hospital readmission, toilet type and maternal employment status.

MUAC, mid-upper arm circumference; HAZ, height-for-age *Z*-score; WHZ, weight-for-height *Z*-score.
