# Supplementary material for: Fat and lean mass predict time to hospital readmission or mortality in children treated for complicated severe acute malnutrition in Zimbabwe and Zambia
Source: Br J Nutr. Author manuscript; Available in PMC 2023 Aug 23. (PMC10442795; doi:10.1017/S0007114522004056)
Supplement: Supplementary Materials [file EMS185001-supplement-Supplementary_Materials.docx]

**SUPPLEMENTARY MATERIAL**

**Fat and lean mass predict time to hospital readmission or mortality in children treated for complicated severe acute malnutrition in Zimbabwe and Zambia**

Mutsa Bwakura-Dangarembizi^1,2,3^, Cherlynn Dumbura^1^, Deophine Ngosa^4^, Florence D Majo^1^, Joe D Piper^1,5^, Jonathan P Sturgeon^1,5^, Kusum J Nathoo^3^, Beatrice Amadi, Shane Norris^2^, Bernard Chasekwa^1^, Robert Ntozini^1^, Jonathan Wells^7^ , Paul Kelly^4,5^ and Andrew J Prendergast^1,5^ for the HOPE-SAM study team.

# Supplementary Fig 1: flow diagram for SAM cohort after hospital discharge

**Discharged 649**

**Missing all measurements at discharge 45**

Number in analysis 604 add to baseline table

**Skinfolds**

Triceps (n= 568)

Subscapular (n=566)

Suprailiac (n=563)

**Bioimpedance**

Phase angle (n=336)

Impedance (Z50) (n= 336)

Reactance (X_c_50) (n=334)

Implausible values n=50

Implausible values n=17

**Sum of skin folds**

Triceps (n= 551)

Subscapular (n=549)

Suprailiac (n=546)

**Bioimpedance**

Phase angle (n=286)

Impedance (Z50) (n=286)

Reactance (X_c_50) (n=284)

**Supplementary Figure 2: Individual trajectories of body composition and anthropometry over 52 weeks of follow up split by HIV status and oedema at initial hospitalisation**

1. **Impedance index Z-score**

*All children HIV negative HIV positive Oedema at baseline No oedema at baseline

**b. Lean mass index Z-score**

*All children HIV negative HIV positive Oedema at baseline No oedema at baseline


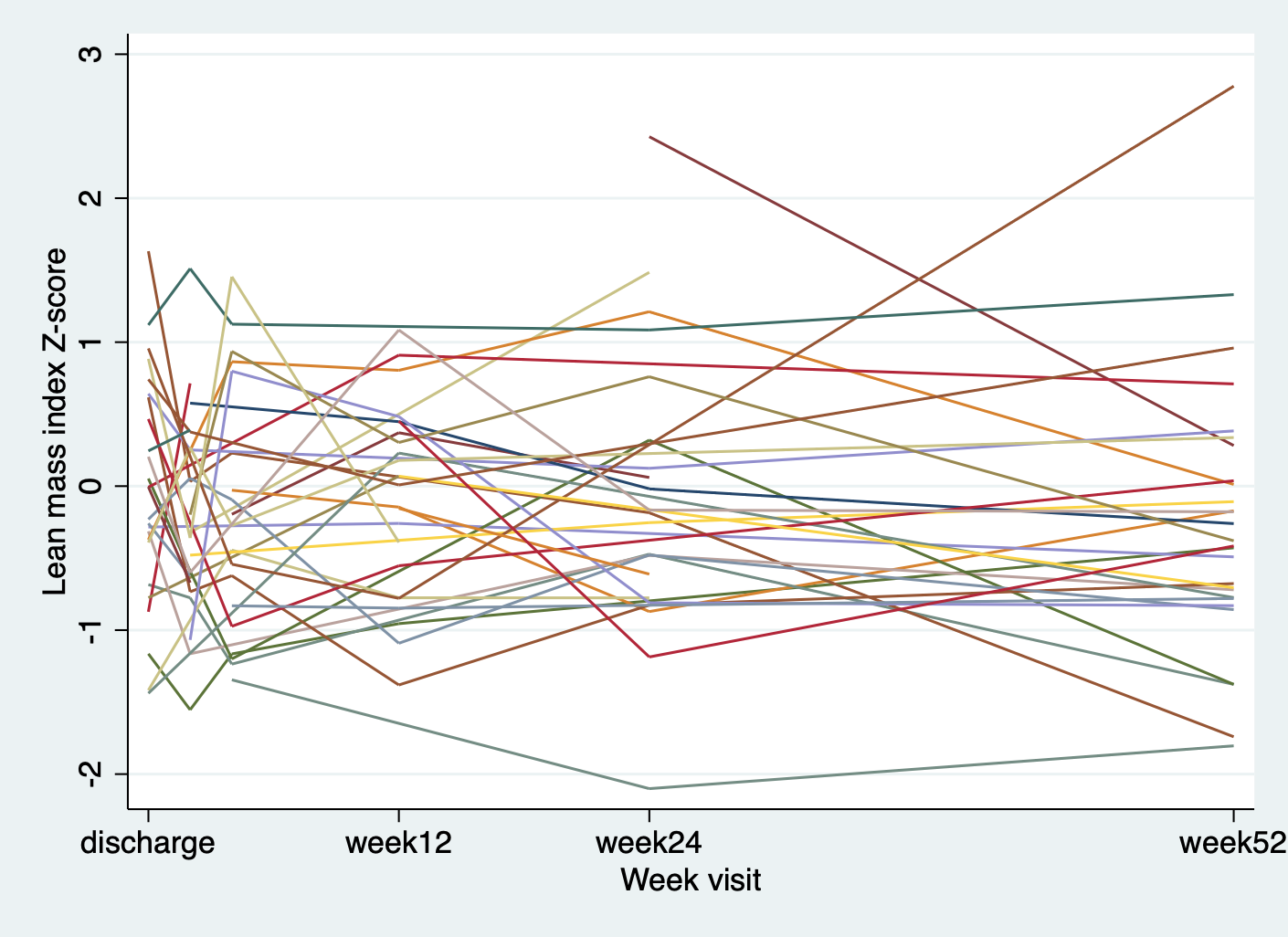

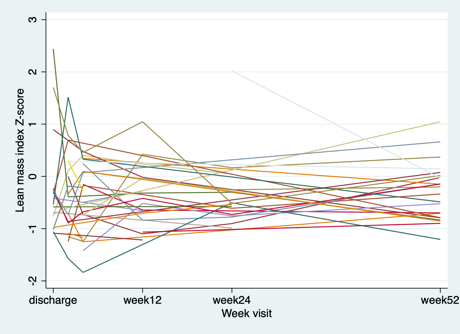

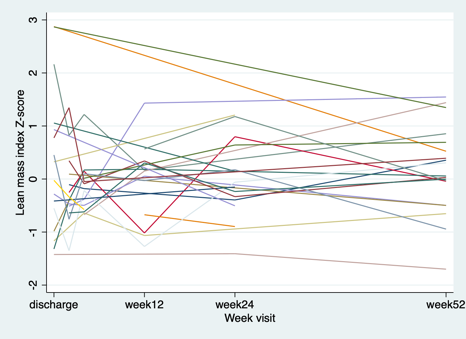

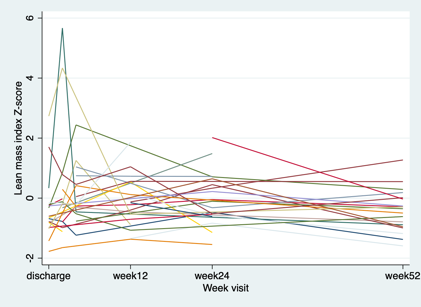


1. **Total skinfolds Z-score**

*All children HIV negative HIV positive Oedema at baseline No oedema at baseline


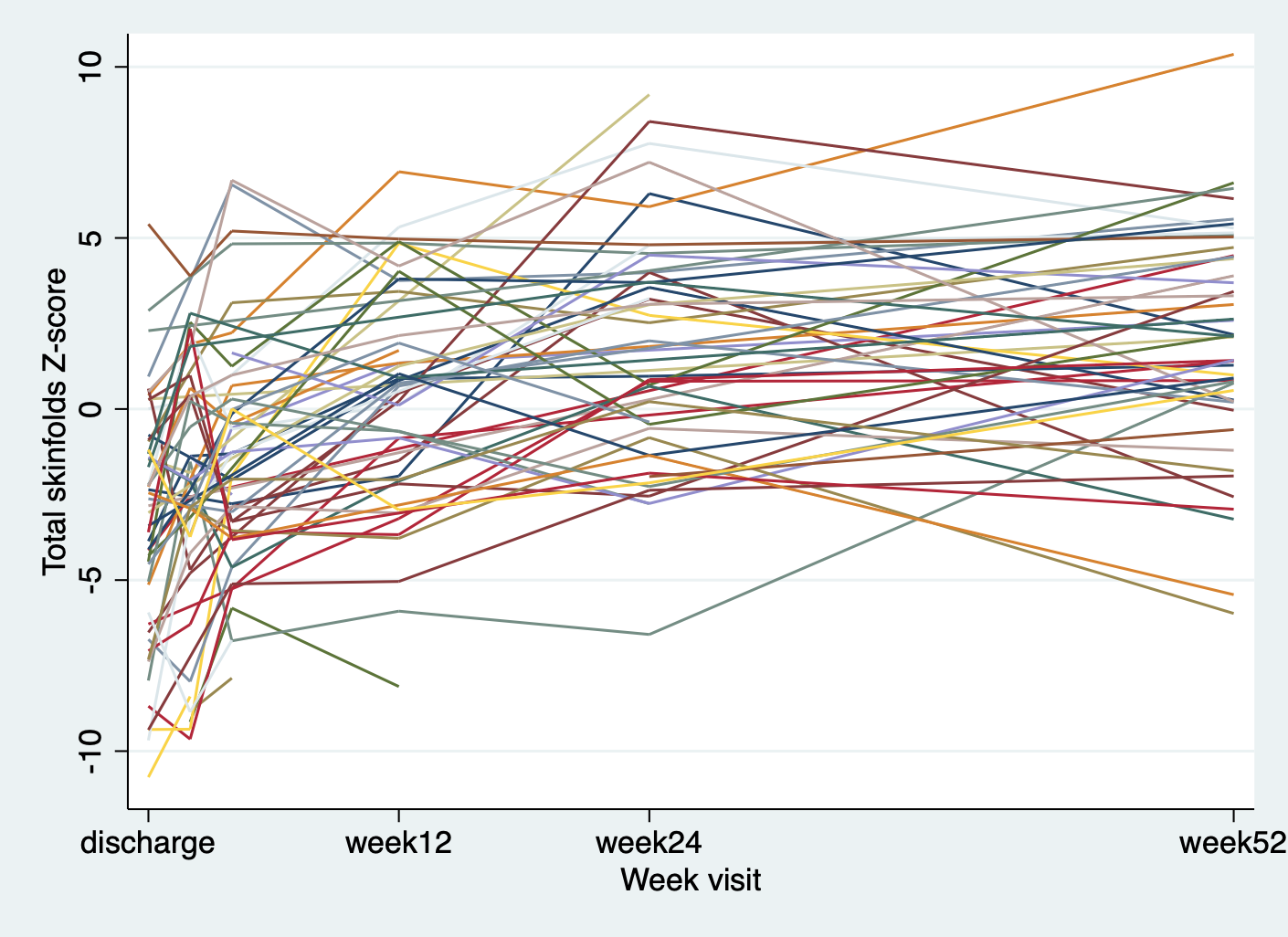

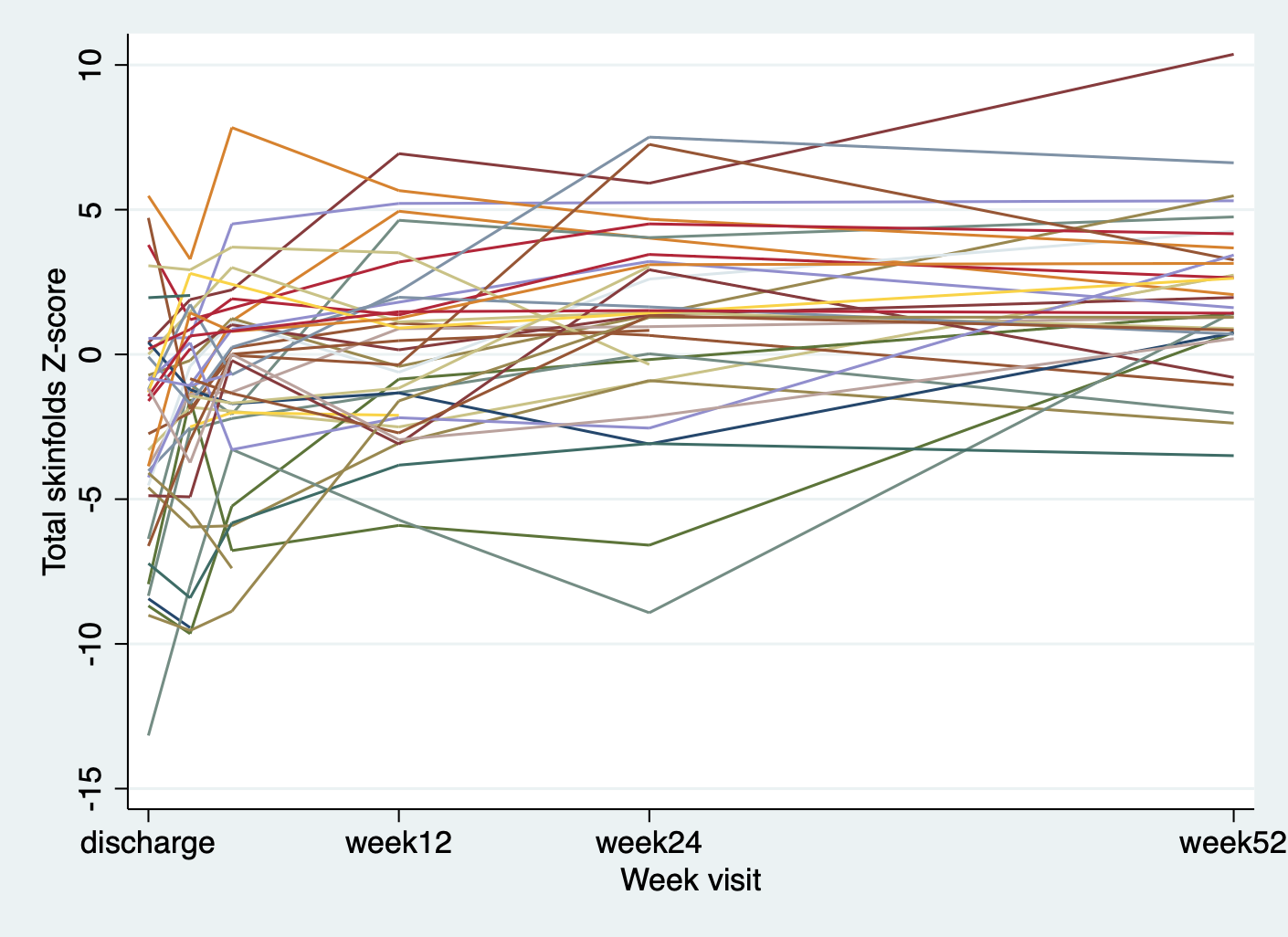

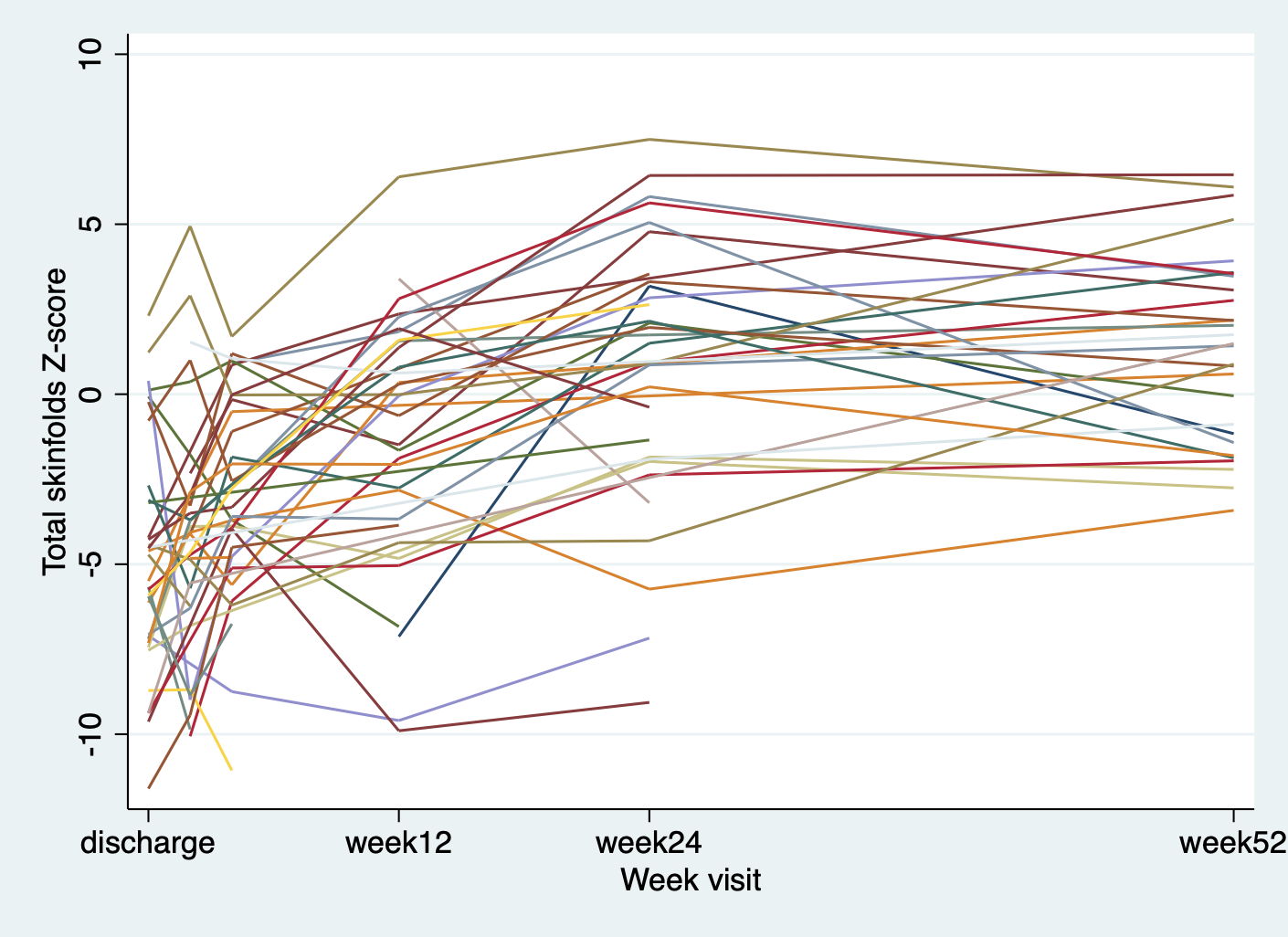

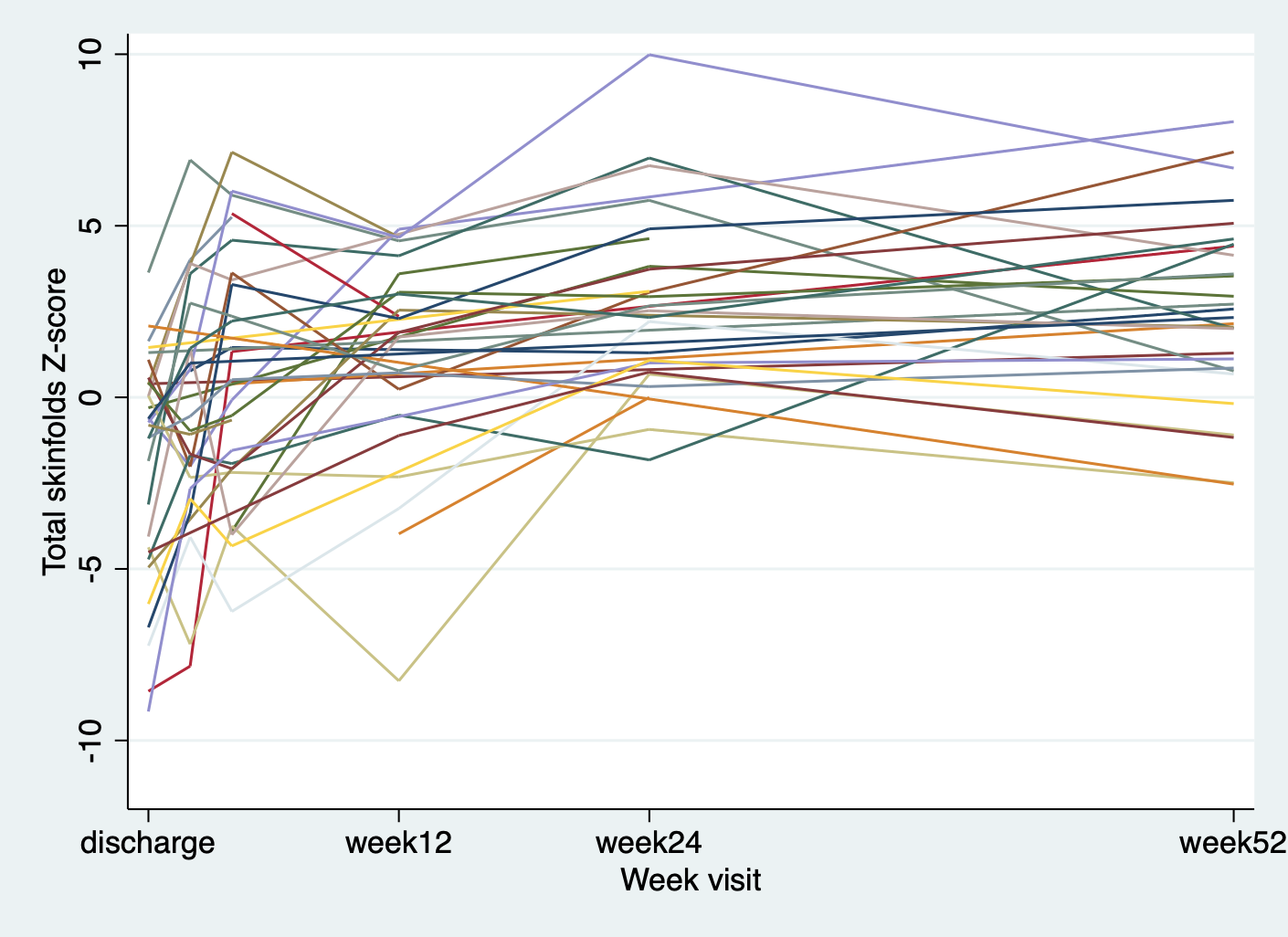

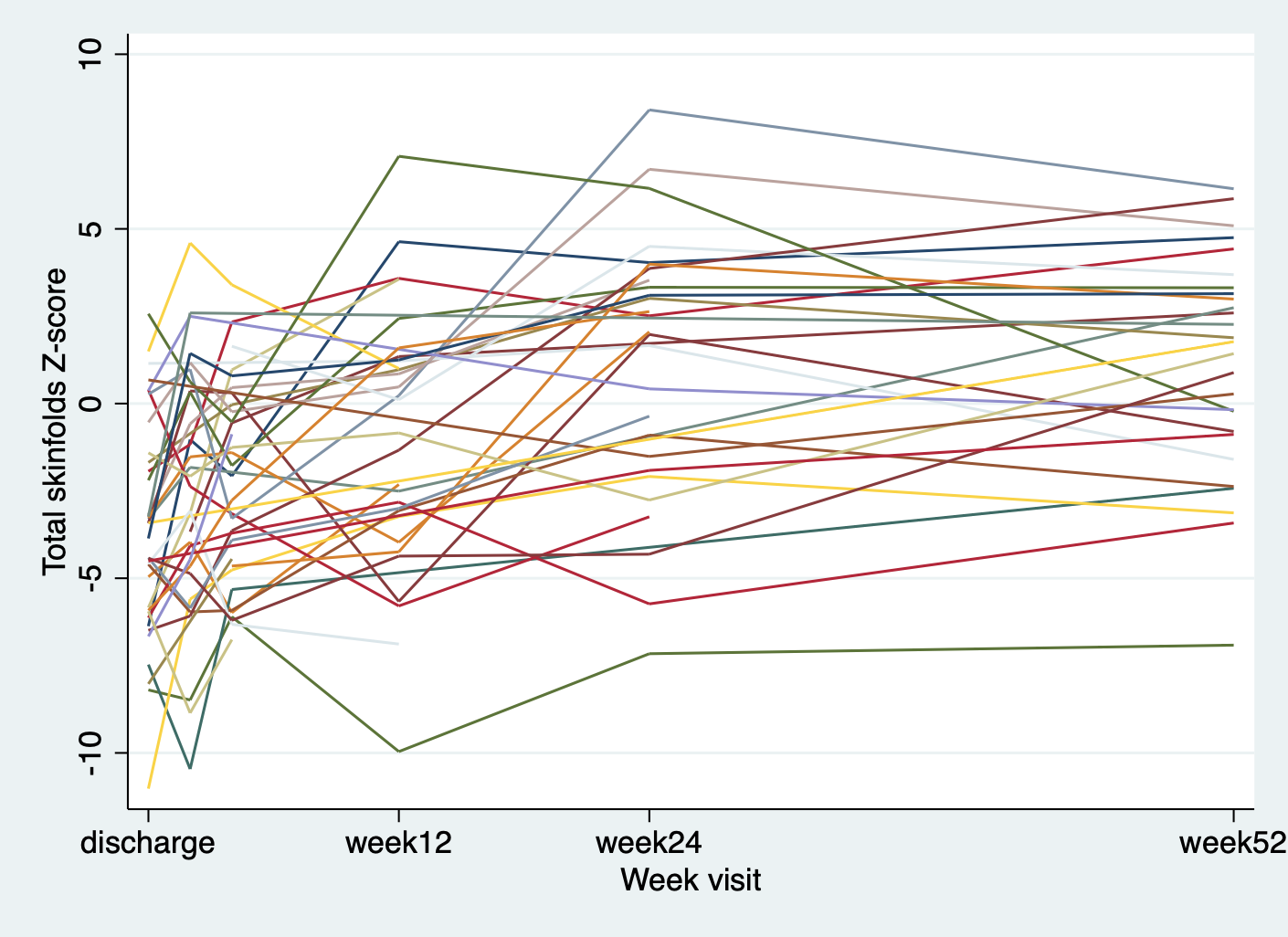


**d. Suprailiac Z-score**

*All children HIV negative HIV positive Oedema at baseline No oedema at baseline


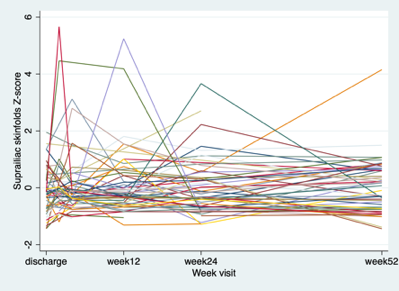

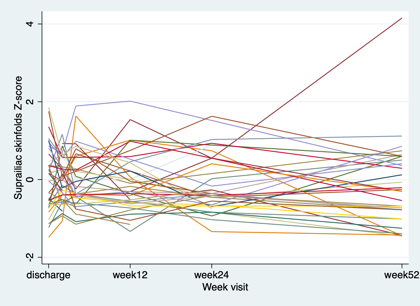

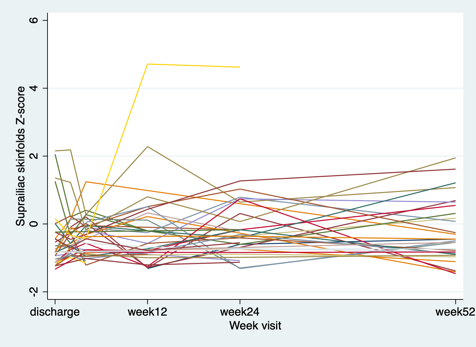

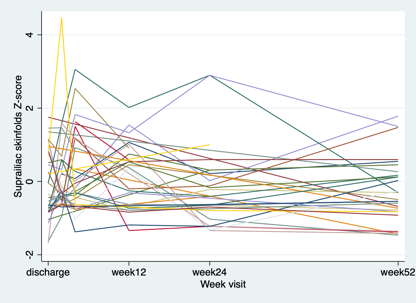

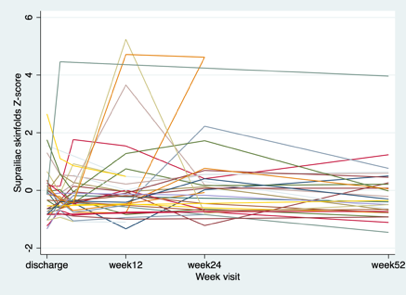


1. **Triceps Z-score**

*All children HIV negative HIV positive Oedema at baseline No oedema at baseline


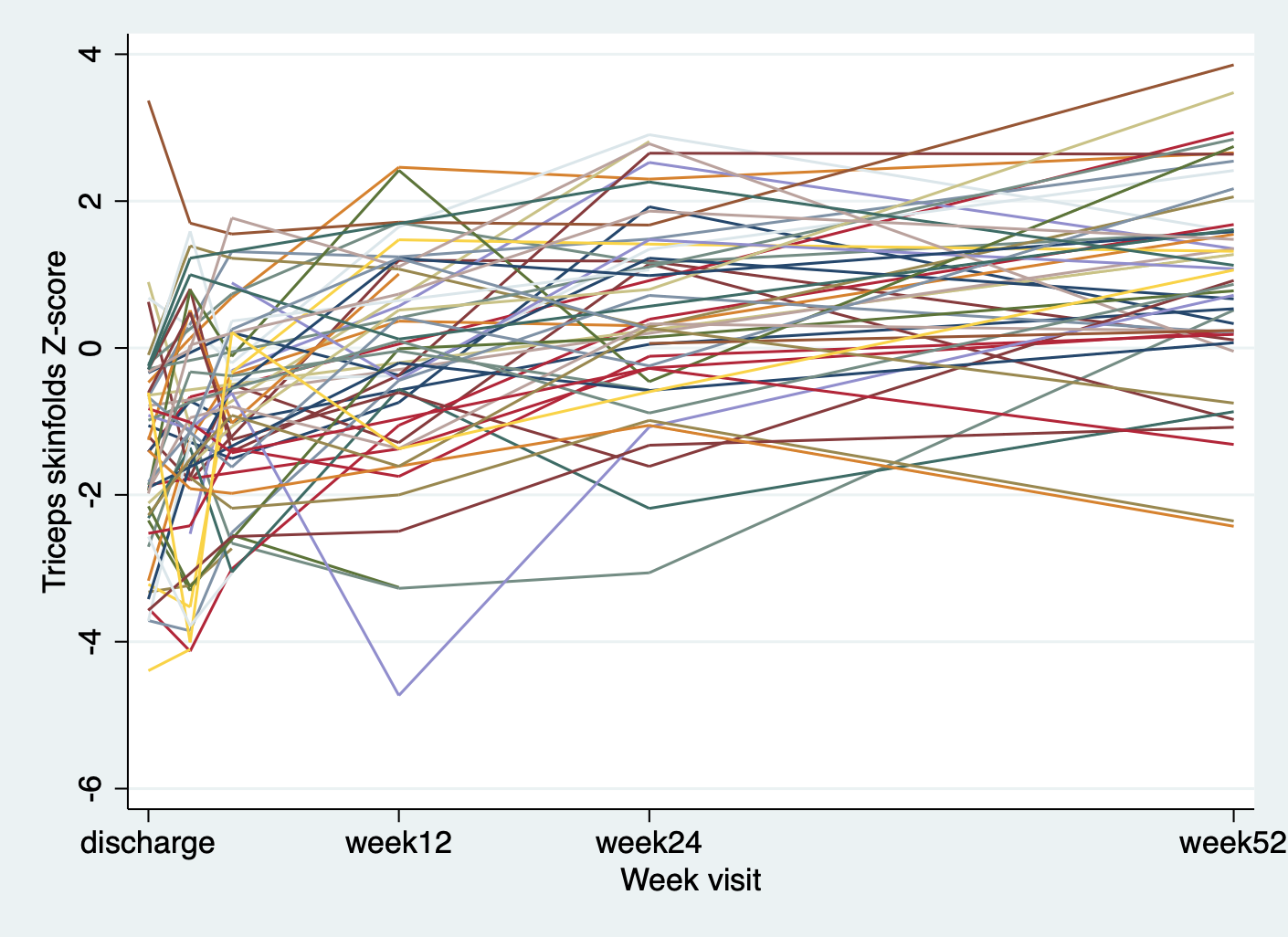

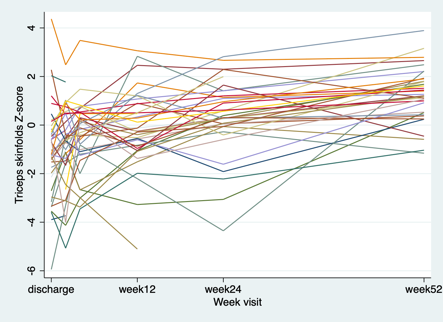

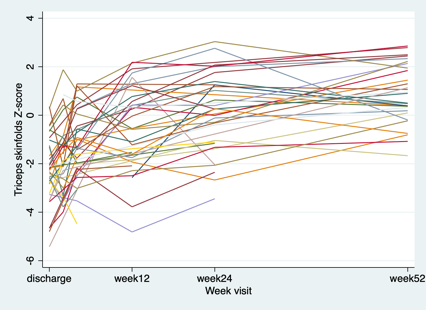

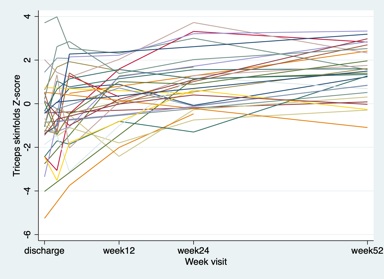

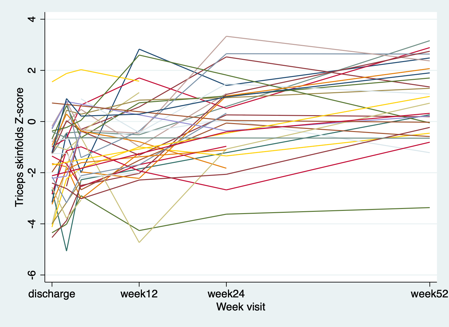


1. **Subscapular Z-score**

*All children HIV negative HIV positive Oedema at baseline No oedema at baseline


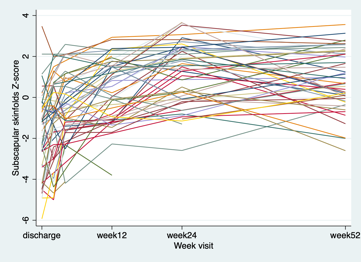

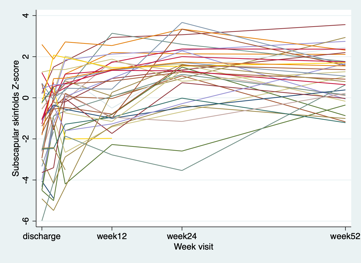

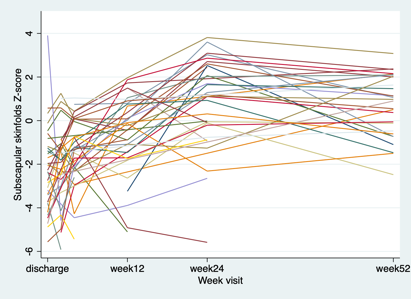

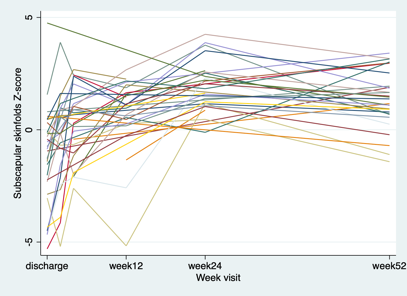

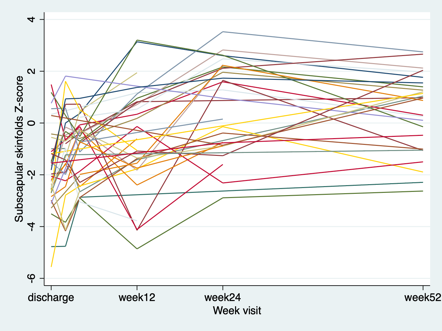


1. **Weight for age Z-score**

*All children HIV negative HIV positive Oedema at baseline No oedema at baseline


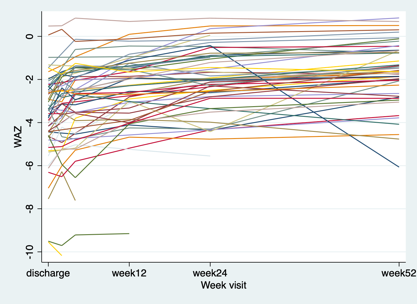

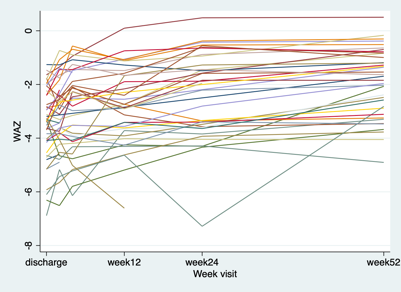

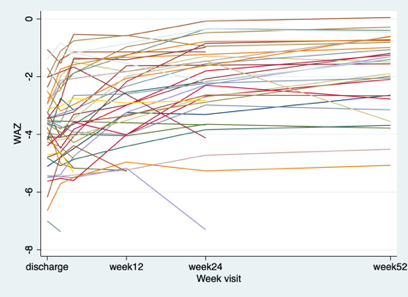

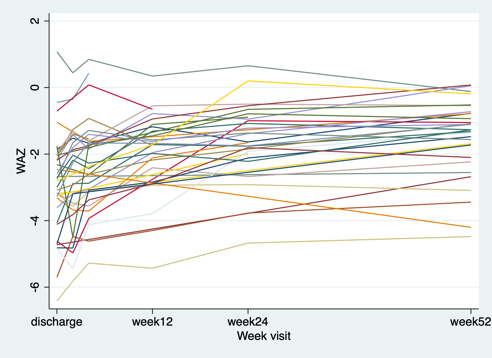

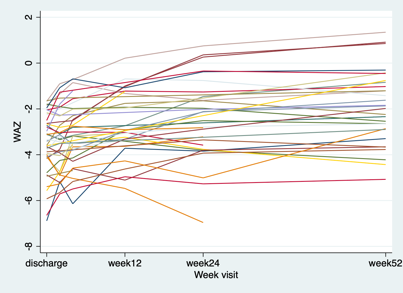


1. **Weight for height Z-score**

*All children HIV negative HIV positive Oedema at baseline No oedema at baseline


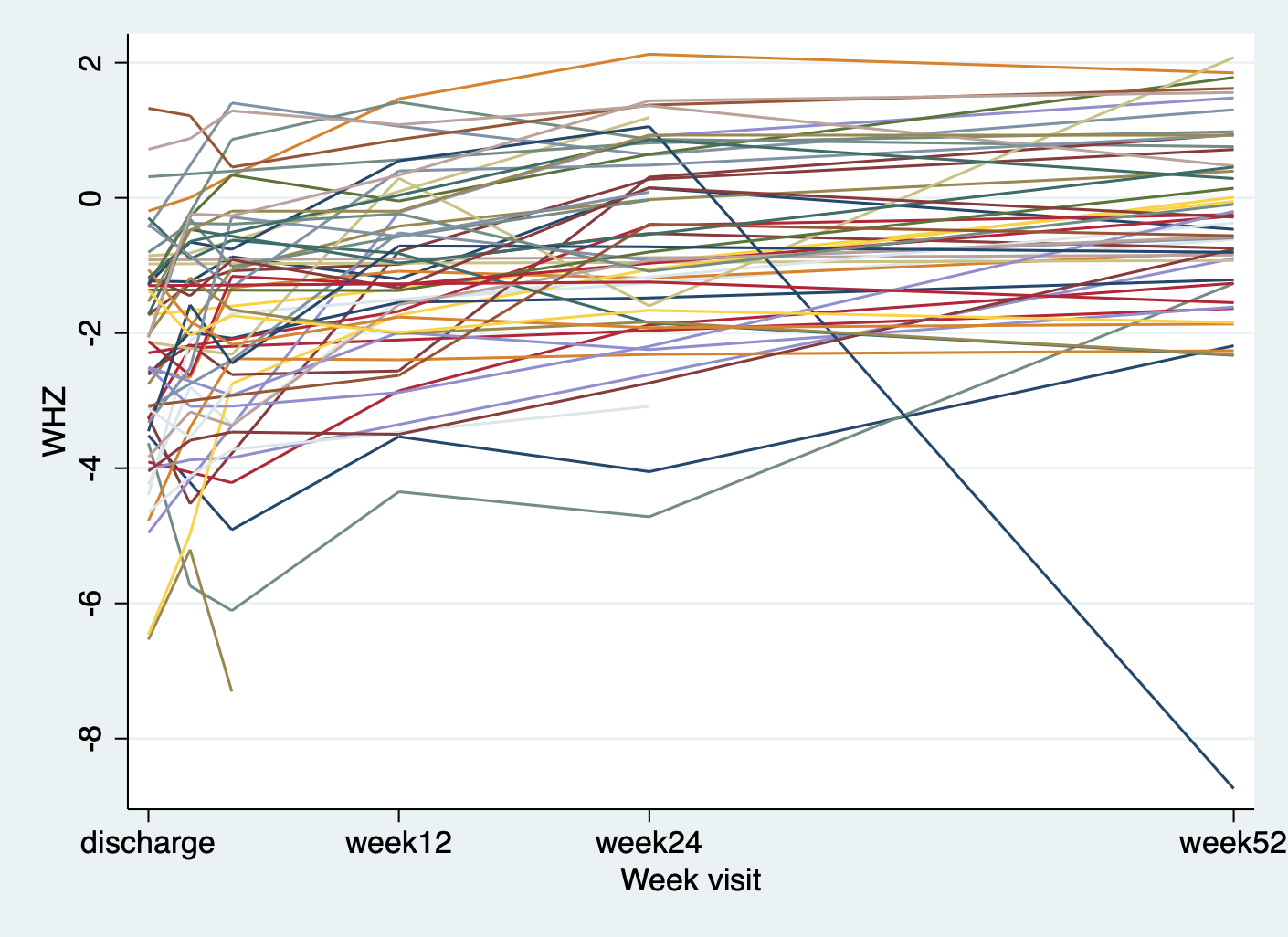

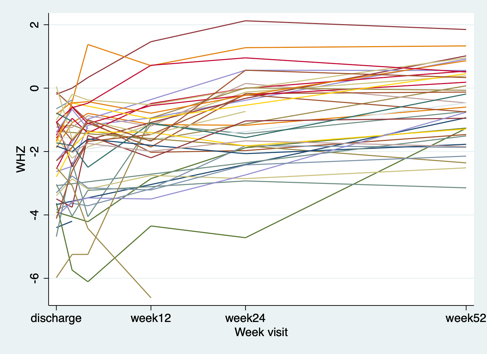

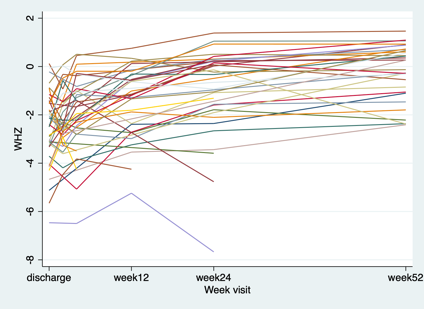

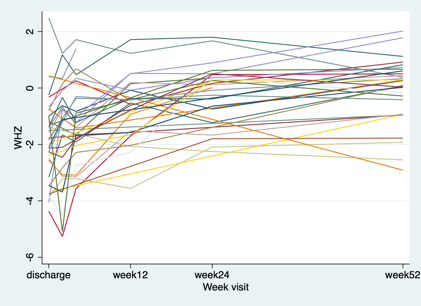

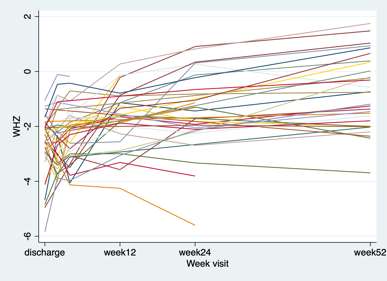


1. **MUAC Z-score**

*All children HIV negative HIV positive Oedema at baseline No oedema at baseline


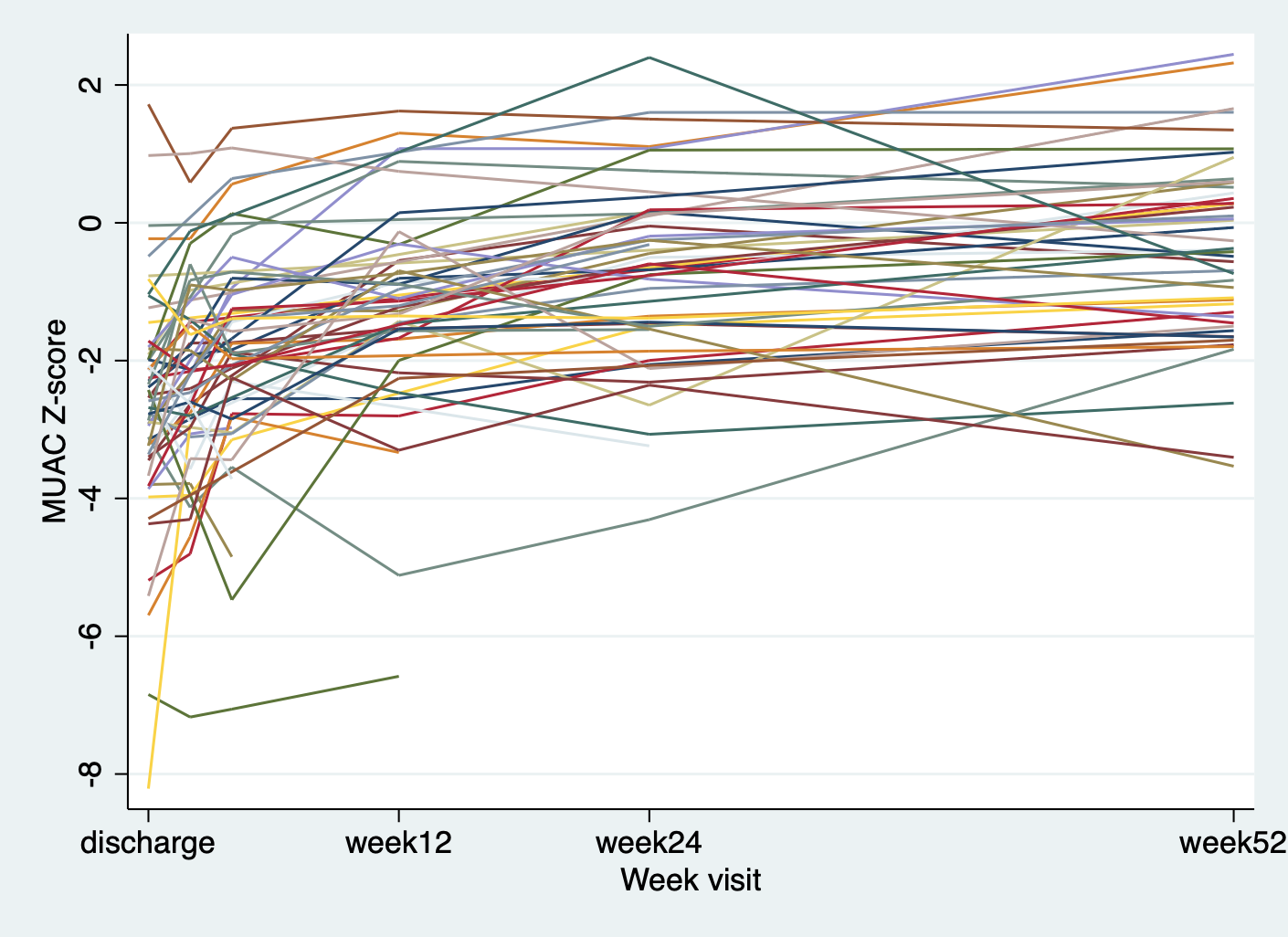

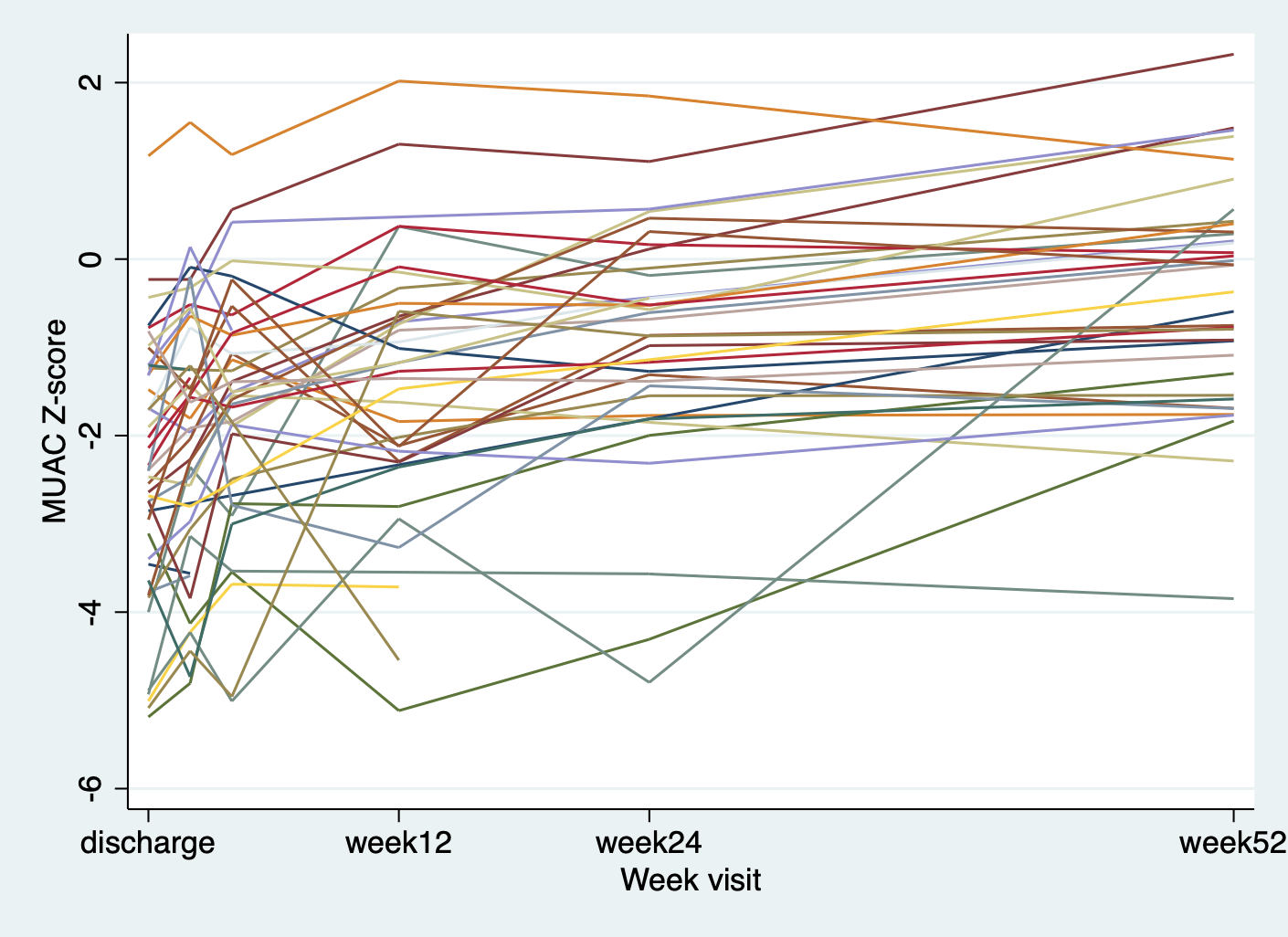

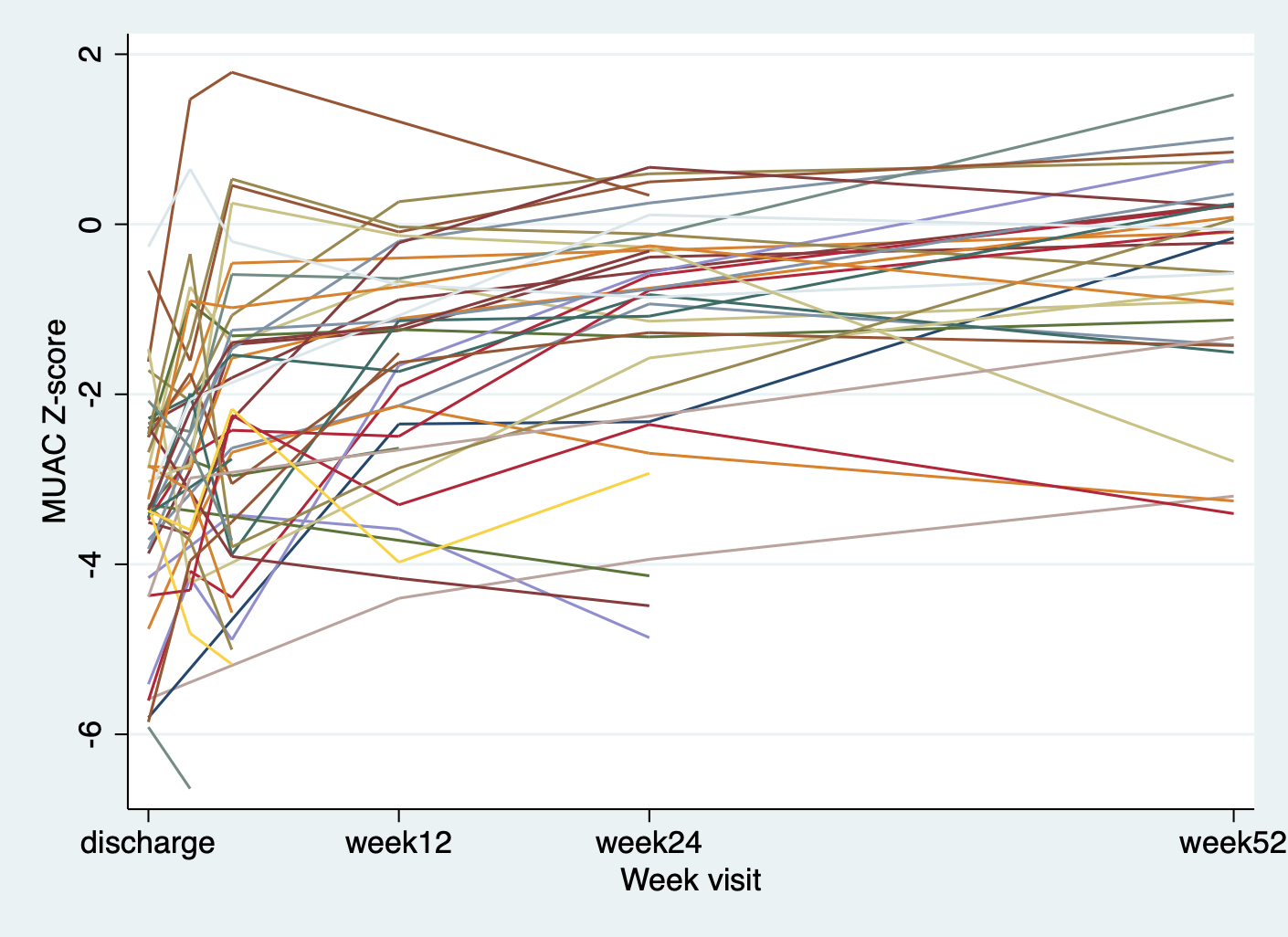

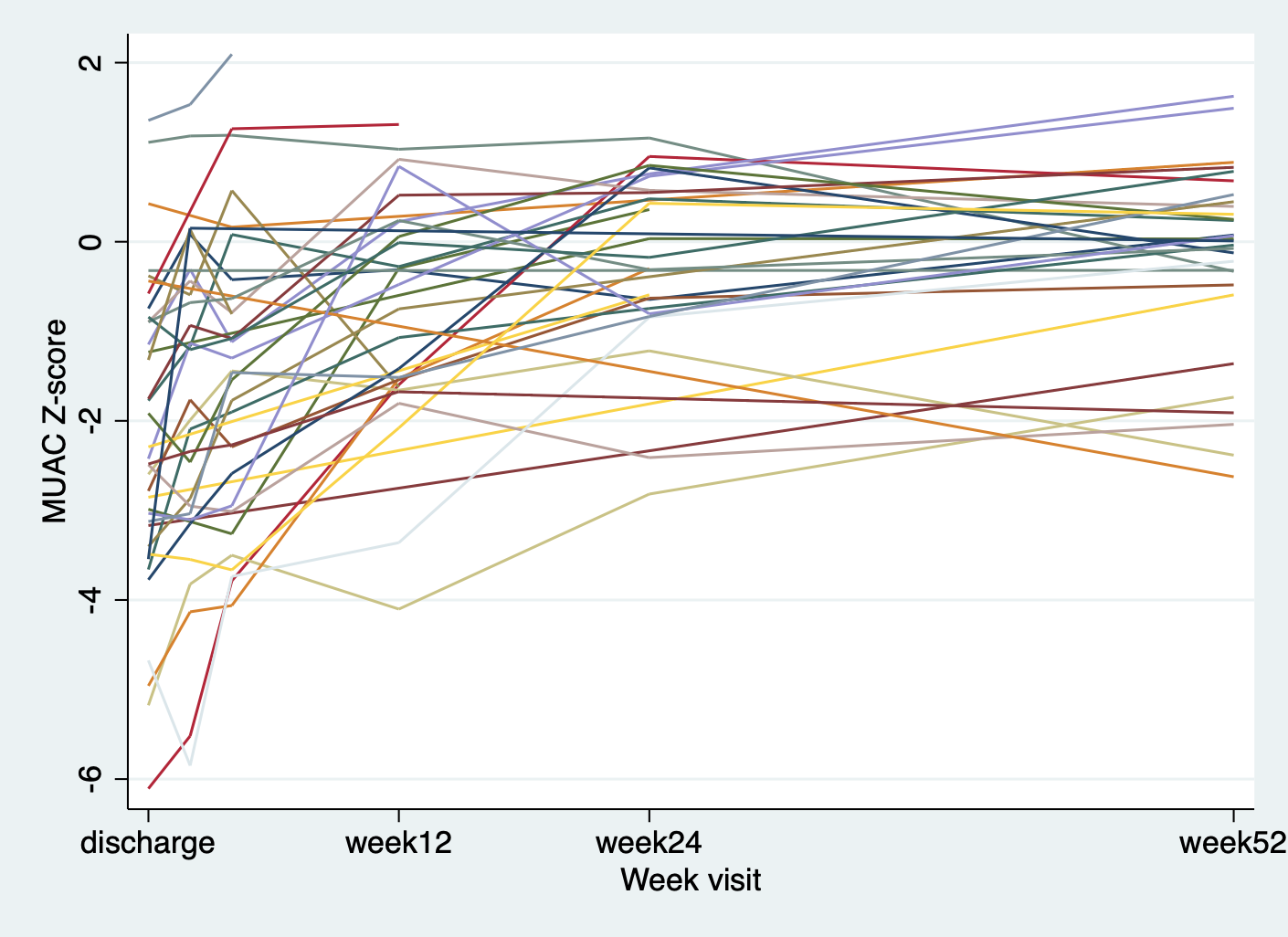

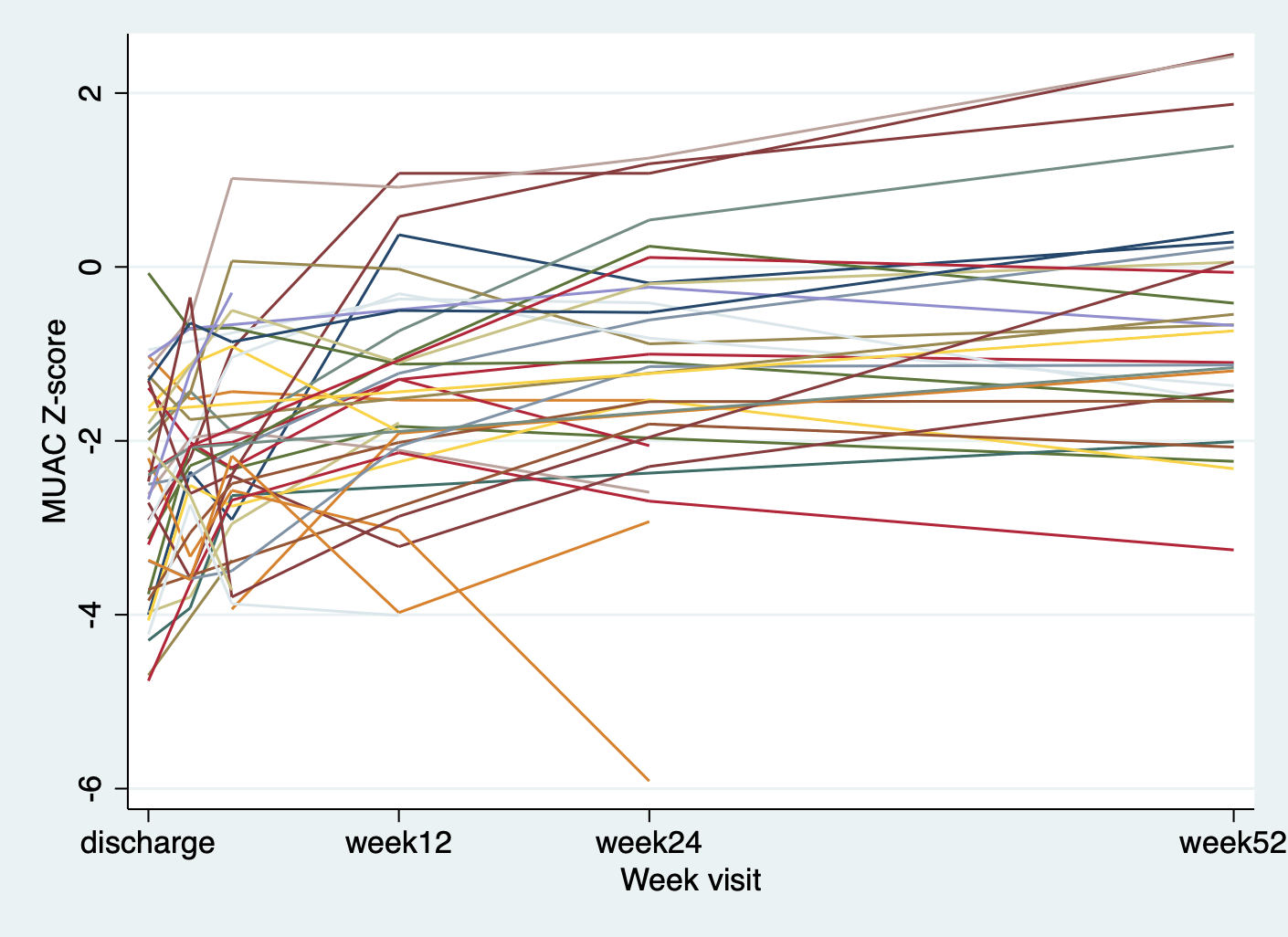


Lowess plots were used to model the changes in body composition and anthropometric variables over the 52 weeks of follow up stratified by HIV status and oedema status at the time of hospitalization. A randomly selected sample of 10% was selected for each variable for the HIV negative and no oedema at baseline due to a large sample. Random samples of 40% and 30% were chosen for the HIV positive and those without oedema because of fewer numbers.

**Supplementary Figure 3: Subgroups of children at discharge identified by anthropometry and body composition variables**

Hierarchical clustering was used to identify groups of children sharing common body composition variables and anthropometric factors that were associated with the outcome of death or readmission. Three distinct groups were identified as shown in Supplementary figure

**Supplementary Fig 4: Direct Acyclic Graphs**

DAGs to identify confounding variables requiring adjustment for the causal relationship between HIV status and oedema at hospitalisation with body composition, and body composition with the composite outcome of death and readmission.

Minimum adjustment set by HIV status: Birth weight, HAZ at discharge, WHZ at discharge, age, baseline oedema, hospital readmission, maternal employment, sex, toilet

Minimum adjustment set by baseline oedema status: Birth weight, age, HIV status, hospital readmission, maternal employment and toilet type


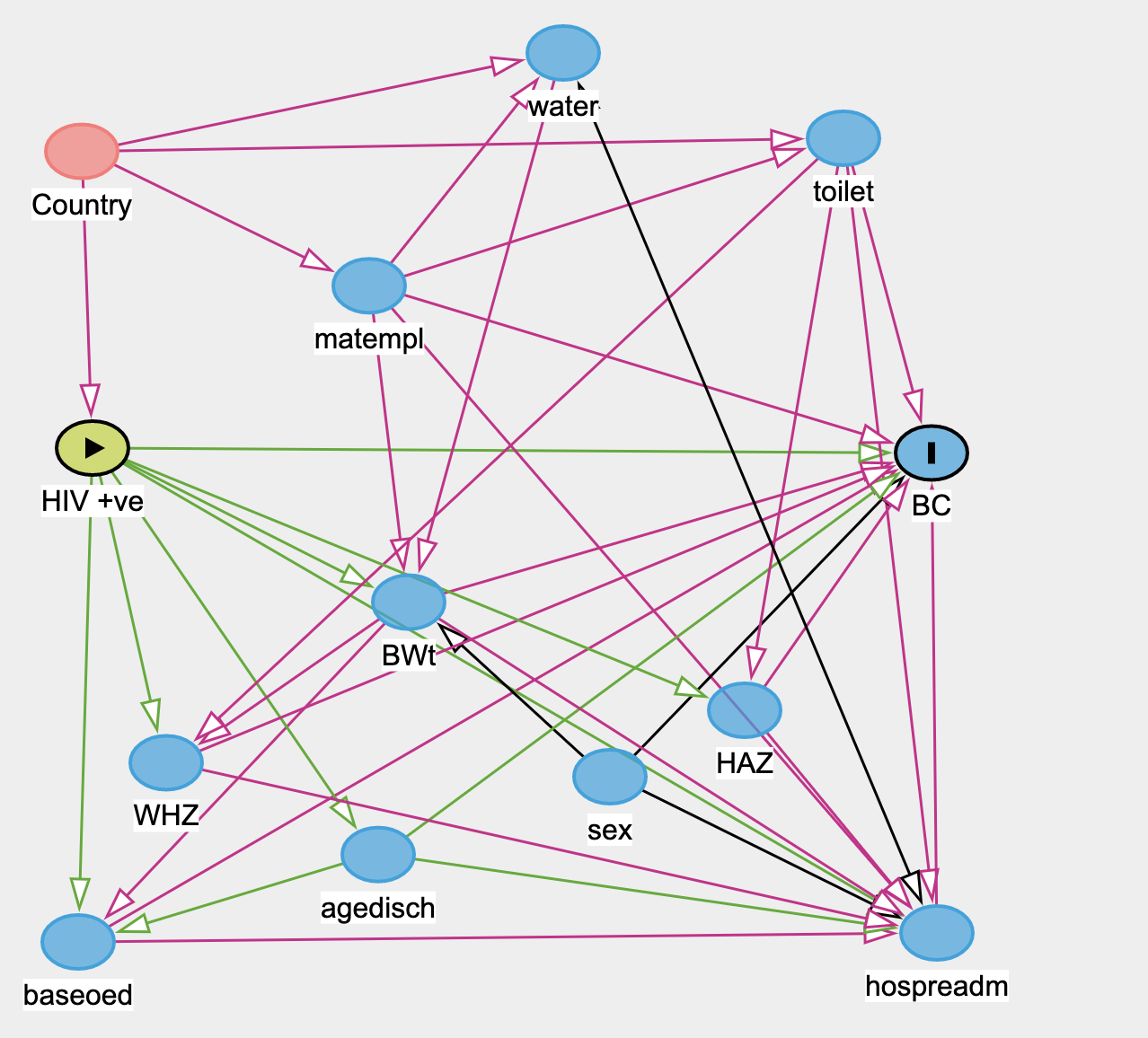

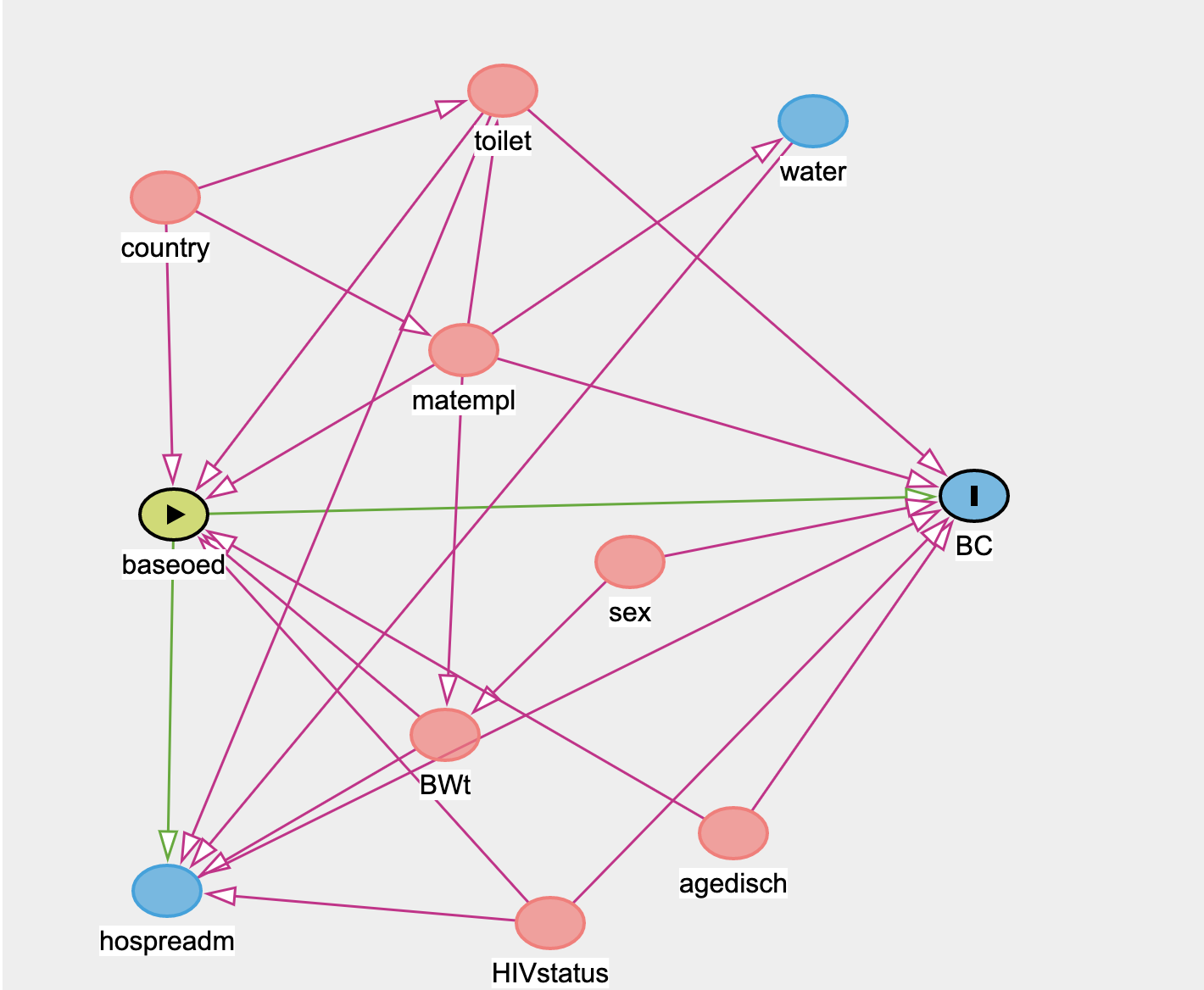


1. Time to event analysis

Minimum adjustment set: age, sex, WHZ at discharge, HAZ at discharge, HIV status, baseline oedema , maternal employment

**
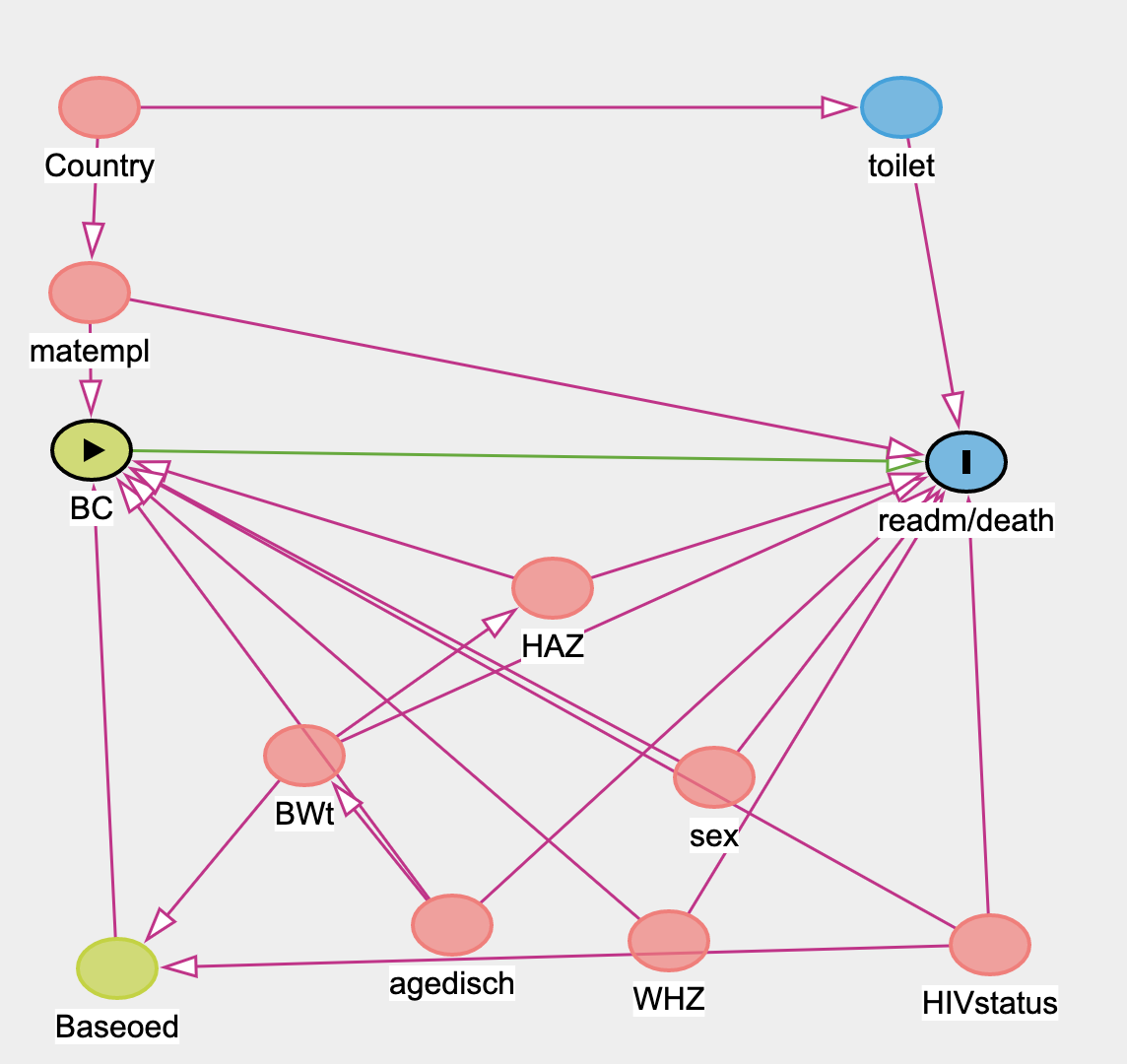
**

**Supplementary Table 1**

**Discharge characteristics of children with and without bioimpedance electrical analysis data**

|  | **No bioimpedance analysis data**  **N=321** | **With available bioimpedance analysis data**  **N=283** | **P-value** |
| --- | --- | --- | --- |
| **Country**   - **Zambia** - **Zimbabwe** | 111/321 (34.6%)  210/321 (65.4%) | 74/283 (26.2%)  209/283 (73.8%) | 0.03 |
| **Male** | 164/321 (51.1%) | 158/283 (55.8%) | 0.24 |
| **Age, months; median (IQR)** | 18 (13, 22) | 19 (14, 24) | 0.09 |
| **HIV status**   - Positive - Negative | 63/321 (19.6%)  258/321 (80.4%) | 58/283 (20.5%)  225/283 (79.5%) | 0.79 |
| **Previous history**   - Complicated SAM - Uncomplicated SAM - Persistent diarrhea - Diarrhea past 2 weeks | 43/317 (13.6%)  62/315 (19.7%)  63/295 (21.4%)  177/321(55.1%) | 47/277 (17.0%)  55/277 (19.9%)  52/261 (19.9%)  149/283(52.7%) | 0.39  0.84  0.66  0.54 |
| Currently breastfeeding  Premature breastfeeding cessation <12mo  ^*^Duration of breastfeeding (months); median (IQR) | 67/319 (21.0%)  90/320 (28.1%)  14 (9, 18) | 51/280 (18.2%)  72/281 (25.6%)  14 (9, 18) | 0.39  0.47  0.81 |
| Oedematous SAM at hospitalisation | 208/321 (64.8%) | 183/283 (64.7%) | 0.97 |
| **SAM at discharge** | 135/321 (42.1%) | 129/283 (45.6%) | 0.38 |
| **Anthropometry**  WHZ, mean (SD)  WAZ, mean (SD)  HAZ, mean (SD)  MUAC, cm; mean (SD) | -2.21 (1.45)  -3.27 (1.52)  -2.96 (1.50)  12.25 (1.60) | -2.18 (1.48)  -3.41 (1.66)  -3.23 (1.57)  12.35 (1.54) | 0.80  0.28  0.03  0.47 |
| Duration of hospitalisation; median (IQR) | 10 (7, 14) | 10 (8, 15) | 0.79 |
| **Chronic underlying conditions**   - Cerebral palsy - Hydrocephalus - Congenital heart disease   **Medications at discharge**   - TB medication - ART medication | 12/321 (3.7%)  2/321 (0.6%)  5/321 (1.6%)  41/321 (12.8%)  29/63 (46.0%) | 15/283 (5.3%)  1/282 (0.4%)  7/282 (2.5%)  42/282 (14.9%)  33/57 (57.9%) | 0.46  0.64  0.420.46  0.19 |
| **Primary caregiver**   - Mother - Age, years; median (IQR)      - Married/stable union - Education, years; median (IQR)   **Employment**   - None - skilled - unskilled   **Residence**   - Rural - Urban - Peri-urban   **Drinking water source**   - Improved   **Toilet facilities**   - Improved - Unimproved - None   **Household electricity** | 291/315 (92.4%)  26 (22, 31)  232/315 (73.7%)  10 (7, 11)  171/314 (54.5%)  30/314 (9.6%)  112/314 (35.7%)  45/319 (14.1%)  195/319 (61.1%)  79/319 (24.8%)  298/315 (94.6%)  280/318 (88.1%)  31/318 (9.8%)  7/318 (2.2%)  150/311 (48.2%) | 256/274 (93.4%)  27 (22, 31)  202/274 (73.7%)  10 (8, 11)  174/273 (63.7%)  12/273 (4.4%)  87/273 (31.9%)  48/282 (17.0%)  173/282(61.4%)  61/282 (21.6%)  261/282 (92.6%)  248/281 (88.3%)  19/281 (6.8%)  14/28 (5.0%)  129/279 (46.2%) | 0.62  0.73  0.98  0.30  0.03  0.48  0.31  0.09  0.63 |

Data are presented as n/N (column percentages) unless otherwise indicated.

# Supplementary Table 2a Cox regression model of discharge body composition factors associated with time to readmission

|  | **N** | **Unadjusted HR (95%CI)** | **P-value** | **Adjusted HR* (95%CI)** | **P-value** |
| --- | --- | --- | --- | --- | --- |
| **Bioimpedance analysis**   - Lean mass index Z-score, - Impedance index Z-score - Phase angle Z-score | N=284  N=286  N=296 | 0.53 (0.37, 0.75)  0.48 (0.33, 0.71)  1.0 3(0.79,1.36) | 0.002  <0.001  0.84 | 0.62 (0.41, 0.94)  0.48 (0.24, 0.83)  1.07 (0.81,1.43) | 0.02  0.01  0.63 |
| **Skin folds**   - Triceps skinfold Z-score - Subscapular skinfold Z-score - Suprailiac skinfold Z-score - Sum of skinfolds Z-score | N= 557  N= 550  N= 563  N= 551 | 0.72 (0.63,0.83)  0.86 (0.76, 0.97)  0.75 (0.58, 0.97)  0.63 (0.47, 0.83) | <0.001  0.01  0.03  0.001 | 0.77 (0.64, 0.91)  0.89 (0.79, 1.02)  0.90 (0.68, 1.19)  0.69 (0.49, 0.98) | 0.002  0.09  0.46  0.04 |

# Supplementary Table 2b Cox regression model of discharge body composition factors associated with time to death

|  | **N** | **Unadjusted HR (95%CI)** | **P-value** | **Adjusted HR* (95%CI)** | **P-value** |
| --- | --- | --- | --- | --- | --- |
| **Bioimpedance analysis**   - Lean mass index Z-score - Impedance index Z-score - Phase angle Z-score | N=284  N=286  N=298 | 0.60 (0.32, 1.02)  0.33 (0.18, 0.60)  0.98 (0.64, 1.49) | 0.06  <0.001  0.92 | 1.01 (0.56, 1.83)  0.74 (0.25, 2.18)  0.91 (0.58, 1.41) | 0.97  0.58  0.66 |
| **Skin folds**   - Triceps skinfold Z-score - Subscapular skinfold Z-score - Suprailiac skinfold Z-score - Sum of skinfolds Z-score | N=557  N=550  N=563  N=551 | 0.68 (0.56, 0.82)  0.88 (0.75, 1.03)  0.49 (0.32,0.73)  0.57 (0.39, 0.85) | <0.001  0.12  <0.001  0.006 | 1.02 (0.80, 1.31)  1.08 (0.93, 1.26)  0.81 (0.53, 1.24)  1.30 (0.85, 2.00) | 0.86  0.31  0.34  0.23 |

*Multivariable models included a minimum adjustment set identified from a directed acyclic graph: age at discharge, sex, baseline oedema, WHZ , HAZ, HIV status, and maternal employment.
